# The main driver of soil organic carbon differs greatly between topsoil and subsoil in a grazing steppe

**DOI:** 10.1002/ece3.9182

**Published:** 2022-08-04

**Authors:** Yantao Wu, Zhiwei Guo, Zhiyong Li, Maowei Liang, Yongkang Tang, Jinghui Zhang, Bailing Miao, Lixin Wang, Cuizhu Liang

**Affiliations:** ^1^ Key Laboratory of Ecology and Resource Use of the Mongolian Plateau Ministry of Education of China Hohhot China; ^2^ Collaborative Innovation Center for Grassland Ecological Security Ministry of Education of China Hohhot China; ^3^ School of Ecology and Environment Inner Mongolia University Hohhot China; ^4^ Department of Environmental Sciences University of Virginia Charlottesville USA; ^5^ Alxa League Meteorological Bureau Bayanhot China; ^6^ Inner Mongolia Meteorological Institute Hohhot China

**Keywords:** carbon fraction, grazing intensity, plant carbon, soil carbon lability, soil depth, soil organic carbon

## Abstract

Soil organic carbon (SOC) dynamics is regulated by a complex interplay of factors such as climate and potential anthropogenic activities. Livestocks play a key role in regulating the C cycle in grasslands. However, the interrelationship between SOC and these drivers remains unclear at different soil layers, and their potential relationships network have rarely been quantitatively assessed. Here, we completed a six‐year manipulation experiment of grazing exclusion (no grazing: NG) and increasing grazing intensity (light grazing: LG, medium grazing: MG, heavy grazing: HG). We tested light fraction organic carbon (LFOC) and heavy fraction organic carbon (HFOC) in 12 plots along grazing intensity in three soil layers (topsoil: 0–10 cm, mid‐soil: 10–30 cm, subsoil: 30–50 cm) to assess the drivers of SOC. Grazing significantly reduced SOC of the soil profile, but with significant depth and time dependencies. (1) SOC and SOC stability of the topsoil is primarily regulated by grazing duration (years). Specifically, grazing duration and grazing intensity increased the SOC lability of topsoil due to an increase in LFOC. (2) Grazing intensity was the major factor affecting the mid‐soil SOC dynamics, among which MG had significantly lower SOC than did NG. (3) Subsoil organic carbon dynamics were mainly regulated by climatic factors. The increase in mean annual temperature (MAT) may have promoted the turnover of LFOC to HFOC in the subsoil. *Synthesis and applications.* When evaluating the impacts of grazing on soil organic fraction, we need to consider the differences in sampling depth and the duration of grazing years. Our results highlight that the key factors influencing SOC dynamics differ among soil layers. Climatic and grazing factors have different roles in determining SOC in each soil layer.

## Graphical abstract

Our results highlight the importance of soil depth and grazing duration in understanding the impact of grazing on SOC. In order to accurately assess the effects of long‐term grazing on SOC content. We suggest that the effects of grazing intensity, grazing duration and climate change on soil biochemical processes should be assessed simultaneously.

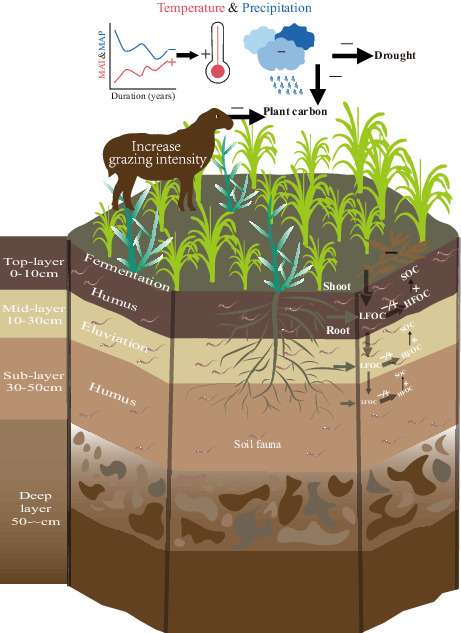

## 1 INTRODUCTION

Grassland ecosystems cover approximately 50 million km^2^ and contain 28%–39% of the terrestrial soil carbon stock (Adams et al., 1990; White et al., 2000); thus, grasslands have been recognized to have great potential for carbon sequestration (Cui et al., 2005). How grassland soil carbon responds to livestock management changes is vital for climate change mitigation and sustainable soil management (Davidson & Janssens, 2006; Paustian et al., 2016). Herbivores play an important regulatory role in grassland ecosystems, controlling the plant and soil carbon balance by foraging and trampling (Frank et al., 2011). However, how herbivores regulate soil carbon dynamics is mechanistically complex (Damien et al., 2015). Understanding the underlying mechanisms of herbivores effects on grassland carbon dynamics is especially important for obtaining sustainable soil management (Liang et al., 2021).

Unfortunately, no consistent relationships between grazing management and carbon sequestration had been reported in the current literature (Wilson et al., 2018). The lack of a clear general relationship between grazing management and carbon sequestration may result from the inconsistent depth of soil sampling within the grassland ecosystems (Ge et al., 2020; Luan et al., 2014). The dynamics of soil organic carbon (SOC) and responses to grazing intensity strongly depended on soil depth (Fontaine et al., 2007). However, few studies have sufficiently quantified the carbon dynamics of different soil layers (Ward et al., 2016). This current gap in knowledge is a major impediment for understanding how grassland ecosystem soil carbon dynamics will respond to changes in grazing intensity with depth. Therefore, a quantitative assessment of the formation of carbon fractions in each soil layer and their response to grazing is essential for our insights into SOC dynamics.

The quantity and rate of SOC turnover are often altered when the grazing management changes (timing, stocking rate, use vs. rest intervals under rotation, etc.). SOC mainly consists of light fraction organic carbon (LFOC) and heavy fraction organic carbon (HFOC) (Zeidler et al., 2002). LFOC largely comprises incompletely decomposed organic residues (Janzen et al., 1992). LFOC responds more rapidly to grazing‐induced changes in the SOC pool than does HFOC (Dubeux et al., 2006; Huo et al., 2013). The dynamics of grazing on the distribution of LFOC in different soil layers may result in an increase or decrease of SOC. As such, researchers have suggested that variations in LFOC could serve as early predictors of SOC changes in the future (Alvarez et al., 1998; Bu et al., 2011; Six et al., 2001). However, previous studies only focused on the dominant effect of grazing on the contribution of carbon fractions to SOC, while ignoring the uncertainties caused by the depth of soil sampling. Thus, it is essential to understand the variations in the different carbon fractions, their current levels, and how they respond to environmental changes and grassland management (Gray et al., 2019).

Knowing how SOC in each soil layer responds to environmental changes and grassland management is vital for climate change mitigation and sustainable soil management (Paustian et al., 2016). The main factors influencing the dynamics of SOC by grazing livestock include (1) climatic variables such as the mean annual temperature (MAT) and mean annual precipitation (MAP); (2) soil conditions including individual SOC fractions; and (3) environmental variables such as soil depth and grazing duration (Luo et al., 2017). Any single focus on the impact of these factors on SOC would lead to great uncertainty (Bradford et al., 2016). The overarching aim of our study was to quantify the effects of long‐term grazing on SOC and soil carbon fractions in different soil layers and to reveal their underlying network relationships. To accomplish this, the following scientific questions were answered: (1) How does increasing grazing intensity and excluding grazing affect the formation and the dynamics between carbon fractions over different soil layers? (2) Does the variation of organic carbon fraction affect the SOC content and stability? (3) Are the effects of grazing and climatic variables on SOC content and stability consistent over soil depths?

## 2 METHODS AND DATA

### 2.1 Study sites

This study was conducted at the Xilin Gol Grassland Nature Reserve, Inner Mongolia Province, China (Figure S1, 44°08′N, 116°19′E, 1129 m a.s.l.). The MAP was approximately 282 mm from 1982 to 2018, of which nearly 85% occurred in the growing season from May to September, which coincided with the peak temperatures (Wang et al., 2018). The MAP of the sampled years was 256 mm for 2014, 309 mm for 2016, and 283.6 mm for 2018 (Figure S2). The soil is classified as Chestnut soil (Chinese soil taxonomic system). The dominant species are *Stipa grandis* P. Smirn (perennial bunchgrass) and *Leymus chinensis* Trin. Tzvel (perennial rhizome grass). Subordinate and transient species include *Cleistogenes squarrosa* Trin. Keng, *Agropyron cristatum* L. Gaertn, and *Carex korshinskyi* Kom.

### 2.2 Experimental design

We fenced off 12 plots (120 × 120 m) in 2011 for this grazing experiment (Figure S3a). Then, we conducted a grazing experiment from 2013 to 2018 using local livestock, which were Inner Mongolian Ujimqin sheep that were approximately 3 years old with a 60 kg body weight. We implemented a completely randomized block design with four grazing intensities and three replicates. The four grazing intensities were defined as no grazing (NG: 0 sheep·ha^−1^ day^−1^), light grazing (LG: 2 sheep·ha^−1^ day^−1^), medium grazing (MG: 4 sheep·ha^−1^ day^−1^), and heavy grazing (HG: 8 sheep·ha^−1^ day^−1^). We implemented sheep grazing in four rounds during the annual growing season each year, from June to September. We adopted the seasonal rotational grazing regime, with each round of grazing lasting 21 days. Grazing started at 07:00 am and ended at 06:00 pm daily, and the sheep had free access to water and minerals. All sheep will be returned to the sheep shelter at the end of each grazing day.

### 2.3 Data sampling

#### 2.3.1 Shoot biomass

The data from all plots were collected in early August in 2012, 2014, 2016, and 2018. Each year, we sampled the transects in each plot, and the data were collected from five quadrats (1 m^2^) along each transect; transects were arranged every 20 m along each plot ~30 m from an eastern fence boundary (Figure S3b). We clipped the residual living shoot tissue of all plants with pruning shears, divided per‐plant species, dried samples to a constant weight at 65°C for more than 48 hours, and then weighed each sample to estimate shoot biomass (g m^−2^).

#### 2.3.2 Root biomass

We used soil cores to measure the root biomass. We harvested root biomass by collecting two 7‐cm diameter soil cores in each quadrat at depths of 0–10, 10–30, and 30–50 cm of each sampled year (2 cores × 3 layers × 5 quadrants × 4 treatments × 3 replicates × 4 years; Figure S3b). First, each sample was packed with a nylon 0.5‐mm mesh bag and shaken by hand under a continuous water flow to eliminate most of the fine soil particles; then, the flotations were collected with a 100‐mesh soil sieve in clean water. All obtained flotations were oven‐dried at 60°C until constant weight to estimate root biomass (g m^−2^).

#### 2.3.3 Soil samples

We collected another soil core in each quadrant at depths of 0–10 cm (top layer), 10–30 cm (mid‐layer), and 30–50 cm (sublayer) to quantify the SOC fraction and nitrogen (1 core × 3 layers × 5 quadrants × 4 treatments × 3 replicates × 4 years; Figure S3b). In the field, each core sample was divided into three layers (top layer, mid‐layer, and sublayer). Before soil carbon analysis, all soil samples were naturally air‐dried and passed through a 2‐mm sieve.

### 2.4 Soil analysis

#### 2.4.1 Soil organic carbon measurements and calculations

We mixed five soil cores from each plot into one composite subsample by soil layers (1 subsample × 3 layers × 4 treatments × 3 replicates × 4 years). Soil total carbon (g kg^−1^) was measured using an automatic element analyzer (Vario MACRO cube, Elementar Analysensysteme GmbH). The soil inorganic carbon content was determined by the gas method with a calcimeter (Calcimeter 08.53, Eijkelkamp) and repeated twice. SOC is the difference between the soil total carbon and the soil inorganic carbon.

#### 2.4.2 Physical fractionation

Twenty‐five grams of air‐dried and sieved soil were placed in 100‐ml centrifuge bottles with 30 ml sodium iodide at a density of 1.8 g/cm^−3^. The bottles were shaken gently by a shock machine (200 r/min) for 1 h and then centrifuged at 5500 rpm for 30 min (Adams et al., 2018). The soil supernatant was removed using a wide‐tipped pipette and placed into 60‐μm nylon mesh bags. The remaining suspension in the bottles was brought back to its initial volume with 30 ml of fresh sodium iodide and re‐centrifuged; then, the residual light fraction was removed. This procedure was repeated no more than twice. The material in the mesh bags was defined as LFOC and rinsed with deionized water 5–6 times. The rinsed samples were weighed after being dried at 40°C and stored in a desiccator until further analysis. HFOC is the difference between SOC and LFOC (HFOC = SOC‐LFOC). The SOC lability is defined as the ratio of labile (LFOC) to nonlabile (HFOC): SOC lability = LFOC/HFOC (Luan et al., 2014).

### 2.5 Statistical analyses

For all exploratory statistics, a critical significance level of *p* < .05 was used. The normal distribution of variances for each data set was tested using the Shapiro–Wilk normality test. To test the effects of the grazing intensity (***GI***), year of grazing (***Y***: 2012, 2014, 2016, and 2018), and soil depths (***D***: top layer, mid‐layer, and sublayer) on SOC, soil carbon fractions (LFOC and HFOC), and plant carbon (shoot biomass and root biomass), repeated measures analysis of variance (ANOVA) was employed using ***GI*** as the between‐subject factor and year and soil depths as a within‐subject factor. Furthermore, to explore the importance of how ***GI*** and year directly altered these plant and soil carbon metrics at different soil depths, we performed repeated‐measures ANOVA using ***GI*** as the between‐subject factor and year as a within‐subject factor. Tukey's‐range test were used to examine differences in plant and soil carbon variables among the grazing treatments.

To explore the plant and soil variables and the correlation relationship, we examined the relationships between shoot biomass, root biomass, total nitrogen (TN), SOC, and SOC lability with Pearson's correlation coefficient across each soil layer. We also fitted a linear model (estimated using ordinary least squares, OLS) and linear ridge regression model to predict SOC (or SOC lability) with grazing duration (Duration), GI, MAP, and MAT (formula: SOC ~ Duration + GI + MAP + MAT). Additionally, to evaluate the SOC explained by each grazing and climate variable in the best model, we employed the averaged over ordering method to decompose *R* ^2^ using the R package *relaimpo* (relative importance).

**FIGURE 1:**
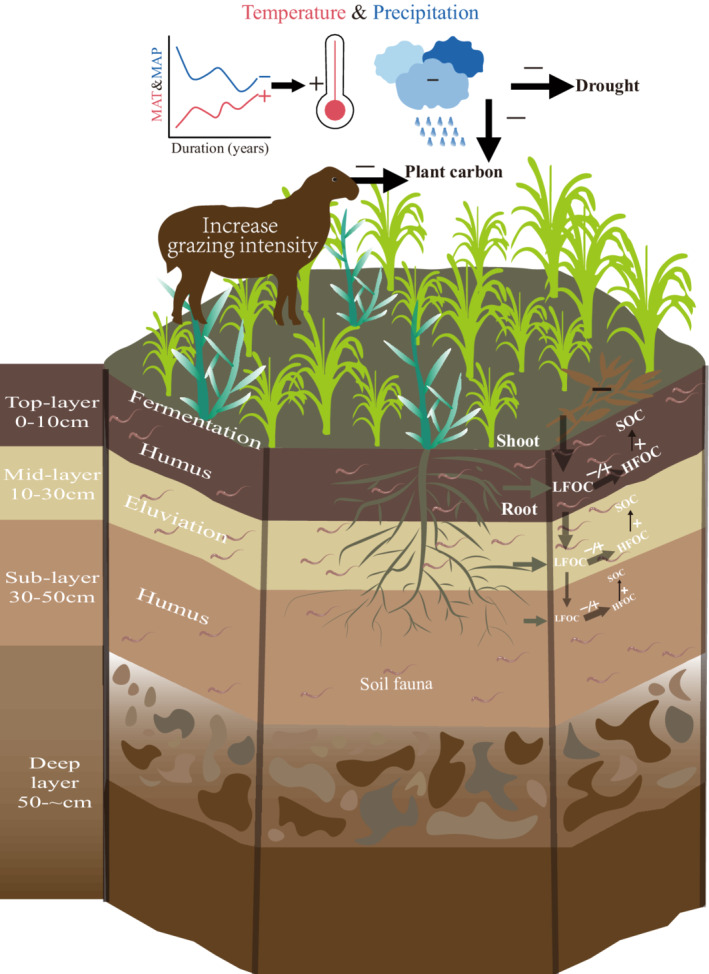
A network framework for the influence of grazing on the SOC across each soil layers in the context of persistent climate warming. There are three key drivers of SOC: the year effects, grazing intensity, and soil depth. Grazing intensity and climate change would decrease shoot biomass and alter the allocation of carbon above and belowground. Shifts in plant carbon input to the soil would further affect SOC. The response of each SOC fraction to grazing and climate may differ with increasing soil depth.

We also performed piecewise structural equation modeling (pSEM) to explore how the grazing‐induced reduction in plant carbon input regulated SOC and SOC stability (Lefcheck, 2016). Our null hypothesis was that the increase in ***GI*** directly regulates the input of plant carbon to soil and indirectly affects SOC lability, thereby affecting the SOC (Figure 1). In this model, to explore only the direct and indirect regulation of grazing on soil and plant carbon, we used linear mixed‐effects models to generate pSEM, in which year was a random factor (formula: SOC~ ., random = ~ 1|year, data). To confirm the final optimal model, we used Shipley's test of d‐ separation with *p* > .05 and chose the pSEM model with the lowest Akaike information criterion (AIC) in the R package *piecewiseSEM*. The conditional ($R_{c}^{2}$) and marginal *R* ^*2*^ ($R_{m}^{2}$) values were calculated for each of the dependent variables. All analyses were conducted in R v4.0.3.

## 3 RESULTS

### 3.1 Grazing effects on plant carbon

**FIGURE 2:**
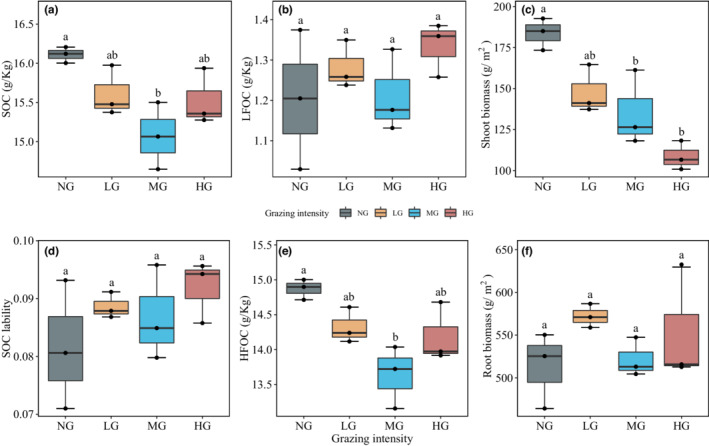
Effect of grazing on multiyear mean of 0–50 cm SOC (a), SOC lability (d), SOC fraction (b: LFOC and e: HFOC), and plant carbon (c: shoot biomass and f: root biomass). Tukey's‐range test were used to examine differences among the grazing treatments, with significant differences marked with different letters (*p* < .05, Mean ± SE, *n* = 3). Key: NG = no grazing, LG = light grazing intensity, MG = medium grazing intensity, HG = heavy grazing intensity.

Overall, grazing significantly reduced shoot biomass, but not root biomass (Table 1). Year significantly affected both shoot and root biomass, while the interaction between year and grazing was also significant (Table 1). There was no significant difference in shoot biomass among treatments before grazing, while there was a significant difference between grazing intensities after grazing treatments (Figure S4a–d). The negative effect of grazing intensity on shoot biomass increased significantly with the increase of years. The dynamics of root biomass were mainly affected by interannual climate fluctuations, with 12.70% (NG), 14.04% (LG), 5.17% (MG), and 17.61% (HG) interannual variation (Figure S4e–h). Compared to the NG treatment, LG, MG, and HG increased the root biomass by 11.47%, 1.63%, and 7.87%, respectively (Figure 2f). Since the interaction effect between grazing and year was weakly significant (Table 1), we were not able to test for significant differences among grazing treatments between each year (Figure S4e–h). Root biomass between soil layers was not significantly affected by grazing intensity, but showed significant interannual variation (Figure S5).

**TABLE 1:** Repeated measures ANOVA results for plant carbon and soil organic carbon fractions using year and soil depth layer as the repeated measures.

Model	DF	*F*‐value	*p*‐value	DF	*F*‐value	*p*‐value
	Shoot biomass (g/m^2^)			Root biomass (g/m^2^)		
Grazing (GI)	**3, 8**	**12.77**	**.002**	3, 8	1.24	.34
Depth (D)	‐	‐	‐	**2, 16**	**342.47**	**<.001**
Year (Y)	**1.32, 13.79**	**100.75**	<**.001**	**3, 24**	**28.19**	<**.001**
GI × D	‐	‐	‐	6, 16	1.21	.35
GI × Y	**5.17, 13.79**	**3.53**	**.006**	**9, 24**	**2.54**	**.03**
Y × D	‐	‐	‐	**6, 48**	**3.41**	**.007**
GI × D × Y	‐	‐	‐	18, 48	1.66	.08
	SOC (g/kg)			LFOC (g/kg)		
GI	**3, 8**	**6.69**	**.01**	3, 8	0.95	.46
D	**2, 16**	**215.59**	<**.001**	**2, 16**	**120.43**	**<.001**
Y	**3, 24**	**3.51**	**.03**	3, 24	2.05	.13
GI × D	6, 16	0.51	.80	6, 16	1.37	.29
GI × Y	9, 24	0.56	.81	9, 24	0.68	.71
Y × D	6, 48	1.65	.15	**6, 48**	**7.16**	<**.001**
GI × D × Y	18, 48	0.90	.58	18, 48	1.39	.18
	HFOC (g/kg)			SOC lability		
GI	**3, 8**	**5.08**	**.03**	3, 8	0.99	.45
D	**2, 16**	**188.90**	<**.001**	**2, 16**	**36.17**	<**.001**
Y	**3, 24**	**4.76**	<**.01**	**3, 24**	**4.55**	**.01**
GI × D	6, 16	0.50	.80	6, 16	1.53	.23
GI × Y	9, 24	0.57	.81	9, 24	0.84	.59
Y × D	**6, 48**	**2.53**	**.03**	**6, 48**	**6.90**	**<.001**
GI × D × Y	18, 48	0.60	.89	18, 48	0.97	.51

*Note*: Sampling was conducted before and after grazing in 2012, 2014, 2016, and 2018*. F* and *p* values indicate ANOVA results and statistical significance, respectively. Values in bold represent a significant difference (*p* < .05 at 95% confidence level, *n* = 3).

### 3.2 Grazing effects on soil carbon fraction and soil organic carbon lability

**FIGURE 3:**
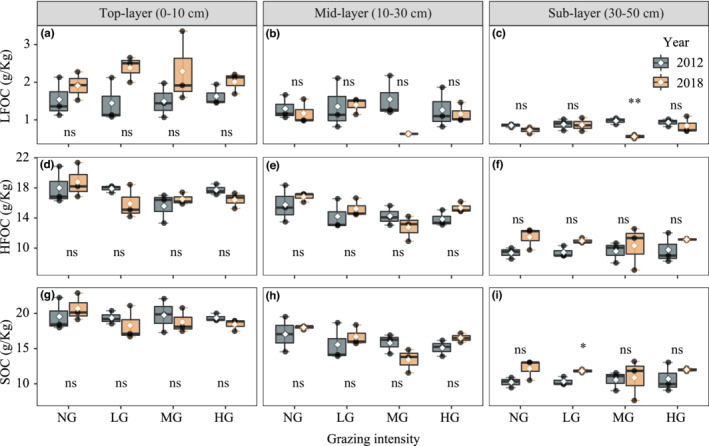
Variation in SOC fraction (a–c: LFOC and d–f: HFOC) and SOC (g–i: SOC) in different soil layers between 2012 and 2018 with different grazing intensities. Independent samples *t‐*tests were used to examine differences between 2012 and 2018, with significant differences marked with * (*p* < .05) and ** (*p* < .01) and nonsignificant differences marked with ns (*p* > .05, Mean ± SE, *n* = 3). Key: NG = no grazing, LG = light grazing intensity, MG = medium grazing intensity, HG = heavy grazing intensity.

**FIGURE 4:**
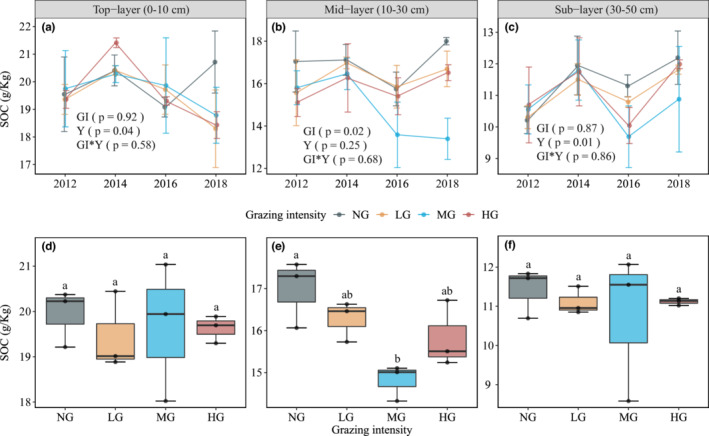
SOC dynamics under different grazing intensities (Mean ± SE, *n* = 3). Shown are the SOC dynamics of the (a) top layer, (b) mid‐layer and (c) sublayer, with statistics (i.e., Tukey's range test) indicating the results from the repeated‐measures ANOVA models of grazing intensity, year and their interactions. Independent samples *t‐*tests were used to examine differences between grazing and nongrazing (d: top layer; e: mid‐layer and f: sublayer), with significant differences marked with * (*p* < .05) and nonsignificant differences marked with ns (*p* > .05). Key: NG = no grazing, LG = light grazing intensity, MG = medium grazing intensity, HG = heavy grazing intensity.

Grazing did not change the LFOC in grassland soils, while it decreased the HFOC and SOC. MG produced a significant effect on SOC and HFOC (0–50 cm depths), while it had no effect on LFOC (Figure 2a,b). Interestingly, LG significantly decreased grassland soil HFOC by ~8.29% compared with NG (Figure 2e). However, LG did not significantly change SOC due to the ~6.57% increase in LFOC (Figure 2a,b). After 6 years of grazing, MG significantly reduced subsoil LFOC, while LG significantly increased subsoil organic carbon (Figures 3 and S6). In addition, grazing significantly changed the SOC and HFOC of the mid‐soil layer, with only MG significantly reducing SOC by 12.75% and HFOC by 12.86% compared with NG (Figures 4 and S7). Finally, the SOC fraction variables at some soil depths showed apparent interannual variations (Figures 4 and S6–7).

**FIGURE 5:**
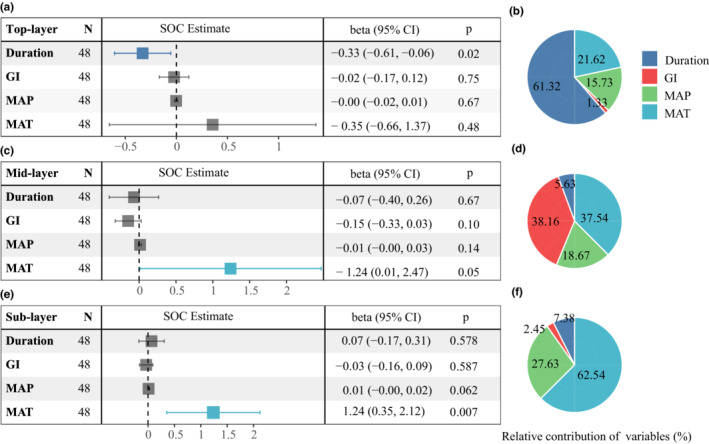
Standardized SOC estimates (a, c, and e) and relative contributions (b, d, and f) of multiple predictors of the ordinary least squares model for SOC in different soil layers (a–b: top layer; c–d: mid‐layer and e–f: sublayer). The standardized effect sizes are shown with their 95% confidence intervals, and relative contributions are assessed using the mean ranking method. Significant differences are indicated by colored square dots (*p* < .05). Duration = grazing duration; GI = grazing intensity; MAP = mean annual precipitation; MAT = mean annual temperature; SOC = soil organic carbon.

Soil organic carbon lability demonstrated significant interannual variabilities at all soil depths (Figure S8). Overall, grazing did not change SOC lability (0–50 cm) in grassland soils, while increasing grazing intensity tended to enhance SOC lability in topsoil (Figure S9a). Years of grazing duration enhanced the response of SOC and SOC lability to grazing. The results of OLS multivariate linear regression and linear ridge regression indicated that years of grazing duration were negatively associated with top‐layer SOC (Figure 5; Table S1) but were not significant in the mid‐layer and sublayer. Interestingly, we found that LFOC in topsoil was negatively correlated with MAP and positively correlated with MAT, yet this relationship was reversed in mid‐soil and subsoil and became stronger with increasing soil depth (Figures S10–S12). Specifically, years of grazing duration was the most important explanatory variable for top‐layer SOC (explaining 61.32% of the variation), while the MAT was the most important explanatory variable for sublayer SOC (explaining 62.53% of the variation). The response of SOC lability to grazing and climate was the opposite (Figure S13). The results of the partial correlation analysis further confirmed the results of the regression analysis (Figure S14). Overall, grazing factors affected the surface soil carbon dynamics, while climate factors affected the subsoil carbon dynamics.

### 3.3 Plant carbon dynamics on soil carbon fraction and soil organic carbon lability

Root biomass determines the vertical distribution of SOC (Figure S15). All plant and soil variables in the random forest model explained 85.8% of the variance in SOC and 74.55% of the variance in SOC lability (Figure S16) Specifically, TN and LFOC were the most important explanatory variables for SOC, followed by root biomass, SOC lability, and shoot biomass (Figure S16). In addition, LFOC was the most important explanatory variable for SOC lability, followed by root biomass, SOC, and shoot biomass. We note that all factors except root biomass significantly affected the variation in SOC lability (Figure S16).

**FIGURE 6:**
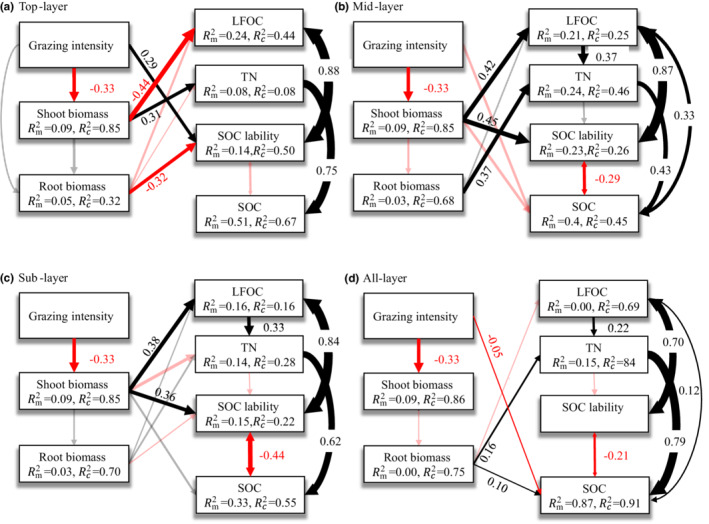
The piecewise structural equation model (pSEM) of grazing intensity predicting the SOC of each soil layers (a: top layer; b: mid‐layer; c: sub layer; d: all layer). The arrows represent unidirectional relationships between variables. Black arrows indicate a positive relationship, while red arrows indicate a negative relationship (significance: *p* < .05). Arrows indicating nonsignificant paths (*p* ≥ .05) are semi‐transparent. The thickness of the significant path is determined by the magnitude of the standardized regression coefficient. $R_{m}^{2}$ and ${\;R}_{c}^{2}$ represent the level of deviance of variables explained by all paths of fixed effects and by fixed and random effects, respectively. A: Fisher's C = 18.04, *p*‐value = .45, df = 18, AIC = 74.04; B: Fisher's C = 11.24, *p*‐value = .67, df = 14, AIC = 71.24; C: Fisher's C = 10.24, *p*‐value = .75, df = 14, AIC = 70.23; D: Fisher's C = 11.42, *p*‐value = .49, df = 12, AIC = 69.42. SOC = soil organic carbon; LFOC = light fraction organic carbon; TN = total nitrogen; $R_{m}^{2}$ = *R* ^2^ marginal; $R_{c}^{2}$ = *R* ^2^ conditional.

Our final structural equation model revealed the pathways and relationships through which grazing influenced grassland carbon cycling in each soil layer (Figure 6). The 85%, 32%, 50%, and 67% conditional variations in shoot biomass, root biomass, SOC lability, and SOC, respectively, were explained by SEM (Figure 6a). However, the pathways and relationships of grazing on plant carbon, SOC fraction, and SOC lability were different in the mid‐layer and sublayer compared with the top layer. ***GI*** directly affected SOC lability in the top layer (Figure 6a) but indirectly affected SOC lability by changing plant carbon and LFOC in the mid‐layer and sublayer (Figure 6b,c). An increase in soil depth increased the negative correlation between SOC and SOC lability. Interestingly, the topsoil LFOC content and shoot biomass were significantly negatively correlated; however, in the mid‐layer and the sublayer, there was a significant positive correlation.

## 4 DISCUSSION

### 4.1 Grazing controls over soil carbon change: carbon fraction

The input of labile carbon (LFOC) may increase the quantity of stable carbon (HFOC) in the soil or may accelerate the decomposition of HFOC (Dijkstra et al., 2021). We found that both grazed and ungrazed LFOC showed a negative correlation with HFOC in the topsoil. Interestingly, we found that grazing shifted this negative correlation to a positive correlation in the mid‐layer and subsoil. This may be due to the fact that microbes are not limited by carbon in the topsoil which allows more HFOC to be decomposed (Soong et al., 2020). However, increasing grazing intensity reduces the input of unstable carbon in the mid‐soil and subsoil, which results in microbes being carbon limited, thus HFOC transformed from LFOC is not readily available to microorganisms.

After six years of grazing and grazing exclusion, we assessed the temporal dynamics of two SOC fractions over different soil layers, when changing ***GI***. Our results showed that grazing exclusion (NG) presented a greater potential to increase the HFOC than grazing (Figure 2e). The multiyear accumulation of litter in NG increased LFOC quantity, led to faster SOC turnover, and resulted in the formation and stabilization of HFOC (Steffens et al., 2009). In addition, there was a tendency for LFOC to increase with GI in the topsoil, which may be due to foraging and trampling by livestock (Figure S6d). The foraging and trampling of livestock promote the physical decomposition of plant residues and contact with the soil, which increases the decomposition rate of litter and transfers carbon to the topsoil (Schuman et al., 2015; Skjemstad et al., 1986). Interestingly, we found that there was a negative correlation between LFOC and shoot biomass in the topsoil and a positive correlation in the mid‐soil and subsoil. This result indicates that the mechanisms of LFOC formation of different soil layers differ significantly. LFOC may accumulate in the subsoil primarily through the activity of macrofauna derived from plant carbon and dissolved organic matter (Zeidler et al., 2002). In summary, we conclude that grazing exclusion may increase LFOC through multiyear accumulation of biomass, while grazing promotes the accumulation of LFOC in the soil by livestock foraging and trampling.

### 4.2 Insights into the mechanisms of grazing on soil organic carbon and soil organic carbon lability

Root biomass contributes significantly to SOC formation compared to shoot biomass (Figure S16a). There is mounting evidence that root‐related carbon input is the most important factor in the formation of SOC (Clemmensen et al., 2013; Sokol et al., 2019). The mean residence time of root‐derived carbon in the soil is 2.4 times longer than that of shoot‐derived carbon due to the higher chemical recalcitrance of root tissue than shoot tissue (Rasse et al., 2005). Thus, root carbon plays a dominant role in the soil carbon pool (Norby & Cotrufo, 1998). However, there were no significant main effects of grazing on root biomass monitored in our study, while root biomass was mainly influenced by interannual variation (Table 1). This finding suggested that SOC content driven by root carbon was mainly influenced by climatic factors. Meanwhile, we found that the interannual fluctuations in root biomass were in the opposite direction to organic carbon. This suggests that SOC driven by root carbon has a significant time delayed effect. This is the reason why we did not test for any correlation between root biomass and SOC in each soil layer (Figure S15).

The SOC is a compound entity consisting of fractions with various residence times on average (Campbell et al., 1967; Debasish et al., 2014). Thus, the concern for the ratios of each carbon fraction in different soil layers can contribute to our understanding of SOC dynamics and the mechanism of carbon sequestration in the presence of different grazing intensities. Accumulation of LFOC in the topsoil caused by increased grazing intensity leads to a decrease in SOC (Figure S6d). This result can be explained by the input of shoot‐derived LFOC that accelerates the decomposition of SOC (Stemmer et al., 1999). In contrast to the topsoil, both the excluded grazing and the increased grazing intensity increased the SOC content of the subsoil, especially under LG. Hence, LG has a stronger carbon sequestration potential than MG and HG in the subsoil (Jiang et al., 2020). In summary, our results suggested that LFOC is an important explanatory factor for SOC. As a labile intermediate fraction, LFOC may be an early indicator of changes in carbon dynamics and total SOC at different grazing intensities (Dong et al., 2021; Six et al., 2002).

SEM showed that the sensitivity of SOC to carbon pool stability increased with the depth of the soil layer. Subsoil organic carbon is conventionally considered to be relatively stable compared to topsoil due to its good insulation in the subsoil (Harrison et al., 2011). However, subsoil organic carbon probably responds more strongly to warmth and grassland management due to its different sources of organic matter, microbial communities, and substrate effectiveness compared to topsoil (Fontaine et al., 2007; Jia et al., 2019; Rumpel et al., 2002). LFOC was the dominant influence on SOC lability in our study. Grazing moderated the quantity of subsoil LFOC mainly by controlling the carbon input from shoot biomass. In turn, the input of fresh plant carbon, represented by LFOC, accelerated the turnover of stable carbon in the subsoil (Fontaine et al., 2007). In summary, the root biomass distribution in each soil layer determined the SOC distribution pattern across the soil profile. However, grazing exclusion and increasing grazing intensity affected the dynamics of organic carbon by shifting the direction of turnover of LFOC to HFOC in each soil layer.

### 4.3 Climatic factors: Interannual variation as a driver of soil organic carbon

In the context of global warming, the typical steppe of Inner Mongolia has experienced a continuous warming process in the last 40 years. Grassland management and climate factors work together to influence the dynamics of SOC (Guo & Gifford, 2002; Luo et al., 2020). Any single focus on the impact of these factors on SOC would lead to great uncertainty (Bradford et al., 2016). ANOVA and SEM both indicated that SOC and organic carbon stability were strongly influenced by MAT and MAP. During our study period, the year of grazing duration (year) was the primary driver of SOC content and lability in the topsoil. Therefore, the duration of the study years may lead to different conclusions. In fact, it is difficult to detect differences in SOC among grazing intensities when the grazing duration is less than 20 years (McSherry & Ritchie, 2013). Grazing and grazing exclusion had a significant timeframe‐dependent effect on the SOC in the topsoil (Luo et al., 2020; Zhang et al., 2018). SOC in the mid‐layer was primarily affected negatively by grazing intensity, showing a decrease mainly in HFOC. In contrast to topsoil, the SOC and SOC lability of subsoil were mainly regulated by the MAT. Partial correlations revealed a strongly significant positive relationship between MAT and SOC, regardless of ***GI*** and duration of grazing. Our results at the local scale differed from those at the global scale (Jobbágy & Jackson, 2000). This difference may be because an increase in MAT in semiarid grasslands with limited precipitation will lead to drought, which would not increase the decomposition of SOC but would instead decrease its decomposition. A warming experiment on the Tibetan Plateau found that raising the temperature of the subsoil would enhance the organic carbon stocks (Jia et al., 2019). Thus, warming in semiarid grasslands may facilitate carbon sequestration in the subsoil (Ding et al., 2017).

## 5 CONCLUSION

When analyzing long time‐series data, the year should not simply be understood as a factor of interannual variability, but should be decomposed into experimental duration (grazing duration) and climatic factors (MAT and MAP). This enables us to accurately assess not only the impact of climatic factors on ecosystem functioning, but also the cumulative effect of grazing over many years. Our results confirm the effects of grazing and climatic factors on SOC and SOC lability in different soil layers. Specifically, grazing time had a significant negative effect on topsoil organic carbon, while subsoil organic carbon was mainly positively influenced by MAT. Due to the large variability of different soil carbon fractions across the soil layers, we propose that SOC dynamics should be assessed separately for each soil layer at different grazing intensities. The duration of grazing or grazing exclusion must be fully considered because there are significant relationships between duration and SOC content and stability in the topsoil. Long‐term continuous monitoring of soil carbon fractions in different soil layers will provide valuable information on how SOC responds to grazing and grazing exclusion in the context of climate change. Current soil carbon and Earth system models are mainly climate‐driven and lack studies of deep soil carbon. To improve the reliability of model predictions, we propose properly incorporating the regulation relationships of different carbon components in each soil layer into carbon models.

## AUTHOR CONTRIBUTIONS

**Yantao Wu:** Conceptualization (lead); data curation (equal); formal analysis (lead); visualization (lead); writing – original draft (lead). **Zhiwei Guo:** Data curation (lead); resources (equal); supervision (lead). **Zhiyong Li:** Investigation (lead); methodology (lead); resources (equal); software (equal); writing – review and editing (equal). **Maowei Liang:** Conceptualization (equal); investigation (equal); methodology (equal); software (equal). **Yongkang Tang:** Investigation (equal); methodology (equal); software (equal); supervision (equal). **Jinghui Zhang:** Data curation (equal); formal analysis (equal); investigation (equal); methodology (equal); writing – review and editing (equal). **Bailing Miao:** Data curation (equal); investigation (equal); methodology (equal); software (equal); writing – review and editing (equal). **Lixin Wang:** Investigation (equal); methodology (equal); supervision (equal); writing – review and editing (equal). **Cuizhu Liang:** Data curation (equal); formal analysis (equal); funding acquisition (lead); project administration (lead); resources (equal); supervision (lead); writing – review and editing (equal).

## CONFLICT OF INTEREST

No potential conflict of interest need be declared by any of the authors.

### OPEN RESEARCH BADGES

This article has earned an Open Data badge for making publicly available the digitally‐shareable data necessary to reproduce the reported results. The data is available at https://doi.org/10.5061/dryad.x69p8czmr.

## Supporting information


**Appendix S1** Supporting Information.

## Data Availability

The data that support the findings of this study are openly available in Dryad Digital Repository at https://doi.org/10.5061/dryad.x69p8czmr.
