# Supplementary material for: The main driver of soil organic carbon differs greatly between topsoil and subsoil in a grazing steppe
Source: Ecol Evol. 2022 Aug 4;12(8):e9182. doi: 10.1002/ece3.9182 (PMC9353232; doi:10.1002/ece3.9182)
Supplement: Supplementary file 1 — Appendix S1 Supporting Information. [file ECE3-12-e9182-s001.docx]

**
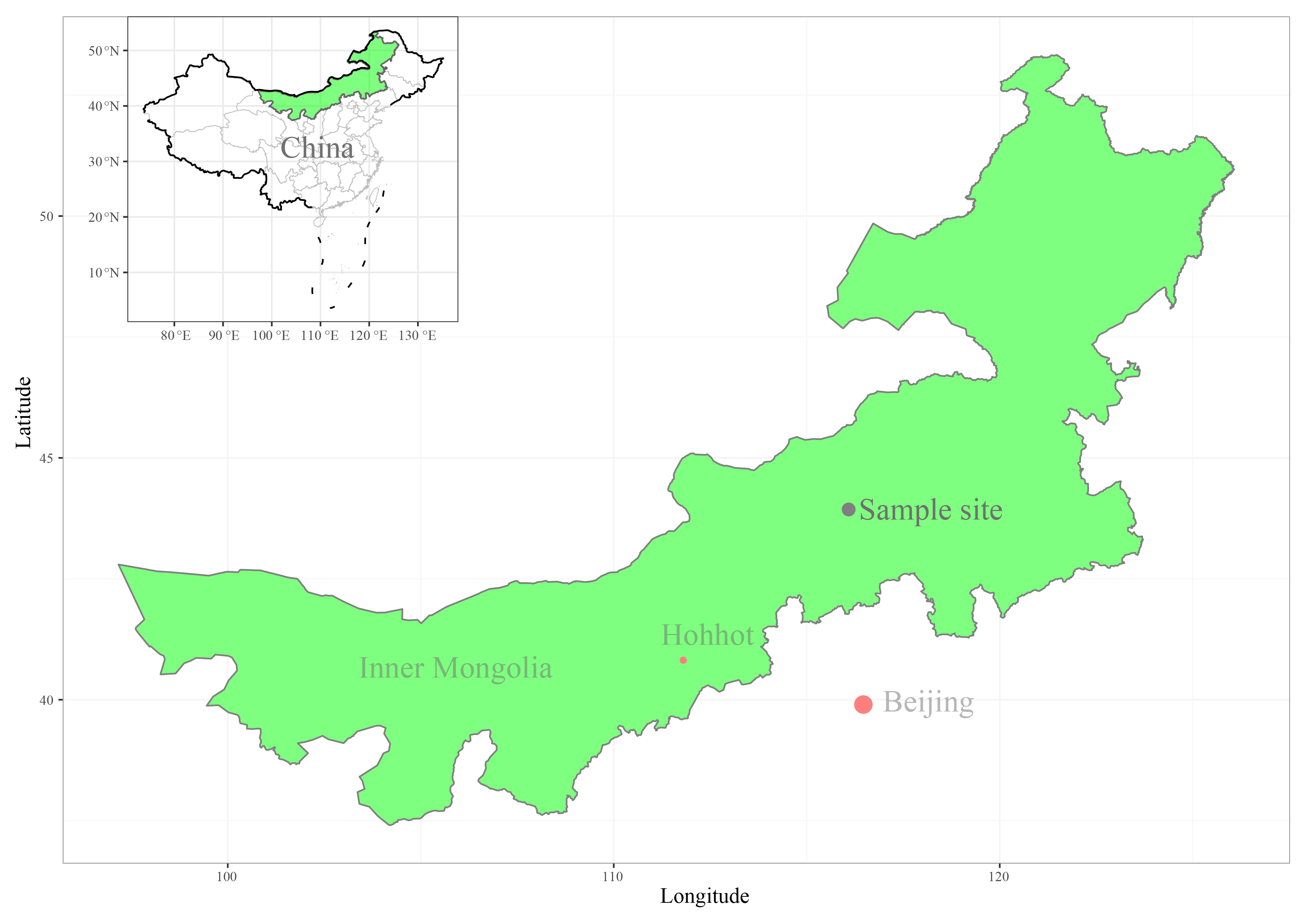
**

**Figure S1.** Geographical location of grazing experimental sample sites.

**
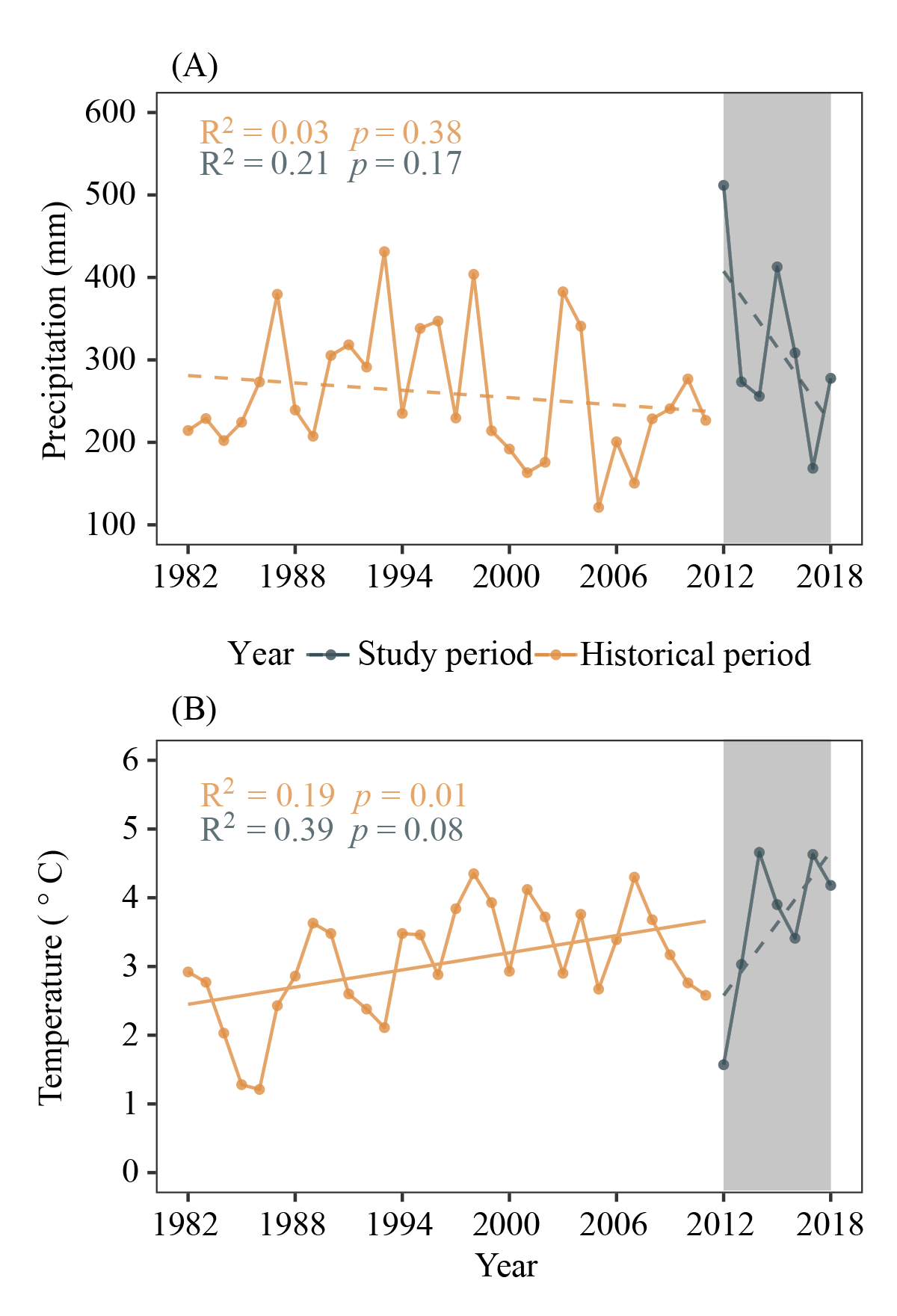
**

**Figure S2.** Mean annual precipitation and mean annual temperature in the study area from 1982-2018. (A): The yellow line shows the precipitation dynamics in historical years, and the grey line shows the annual precipitation dynamics in the study years. (B): The yellow line shows the temperature dynamics in historical years, and the grey line shows the annual temperature dynamics in the study years.


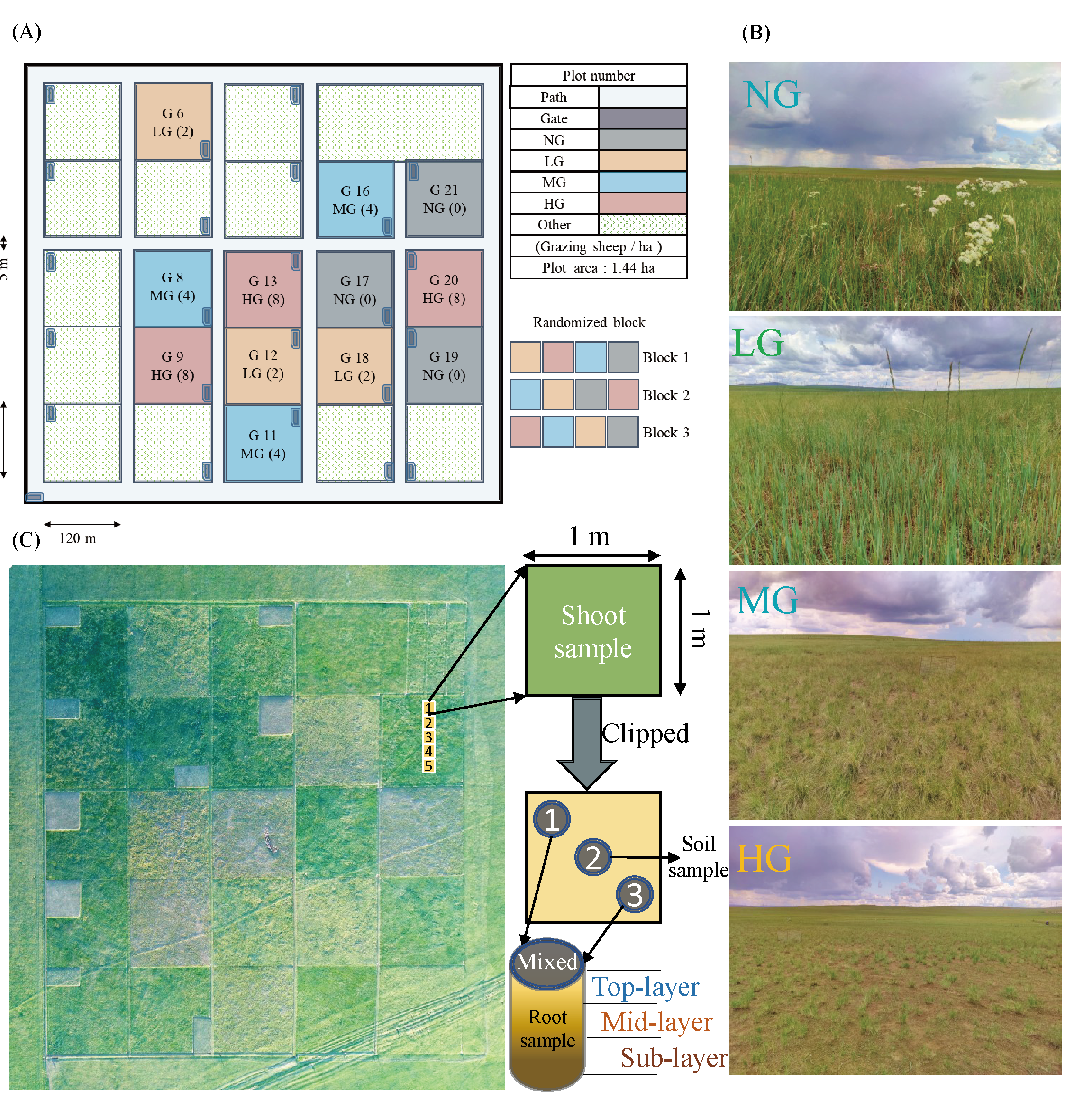


**Figure S3.** Experimental design and landscape. (A): The randomized block grazing experiment was designed with four treatments: light grazing (LG: 2 sheep·ha^−1^·day^−1^), medium grazing (MG: 4 sheep·ha^−1^·day^−1^), heavy grazing (HG: 8 sheep· ha^−1^·day^−1^) and no grazing (NG: 0 sheep·ha^−1^·day^−1^). Each plot is 1.44 ha. (B): The landscape of the four grazing treatments. (C): Aerial view of the grazing experimental platform and sampling scheme.


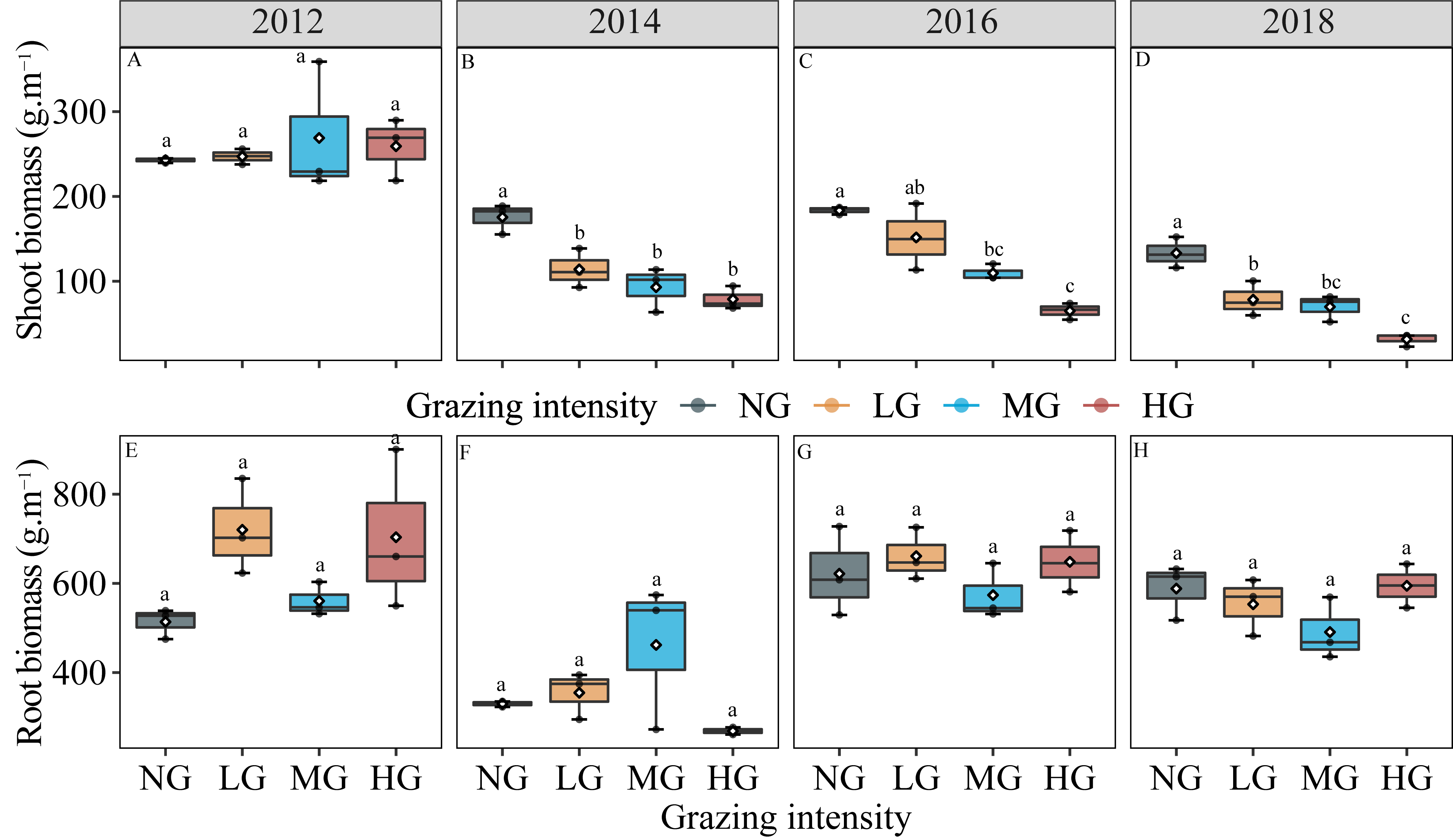


**Figure S4.** Shoot biomass (A-D) and root biomass (E-H) varied in different years under different grazing intensities. Shown are the results of Tukey’s-range test among different grazing intensities. Lowercase letters represent significant differences among grazing treatments (*p* < 0.05). Key: NG = no grazing, LG = light grazing intensity, MG = medium grazing intensity, HG = heavy grazing intensity.

**
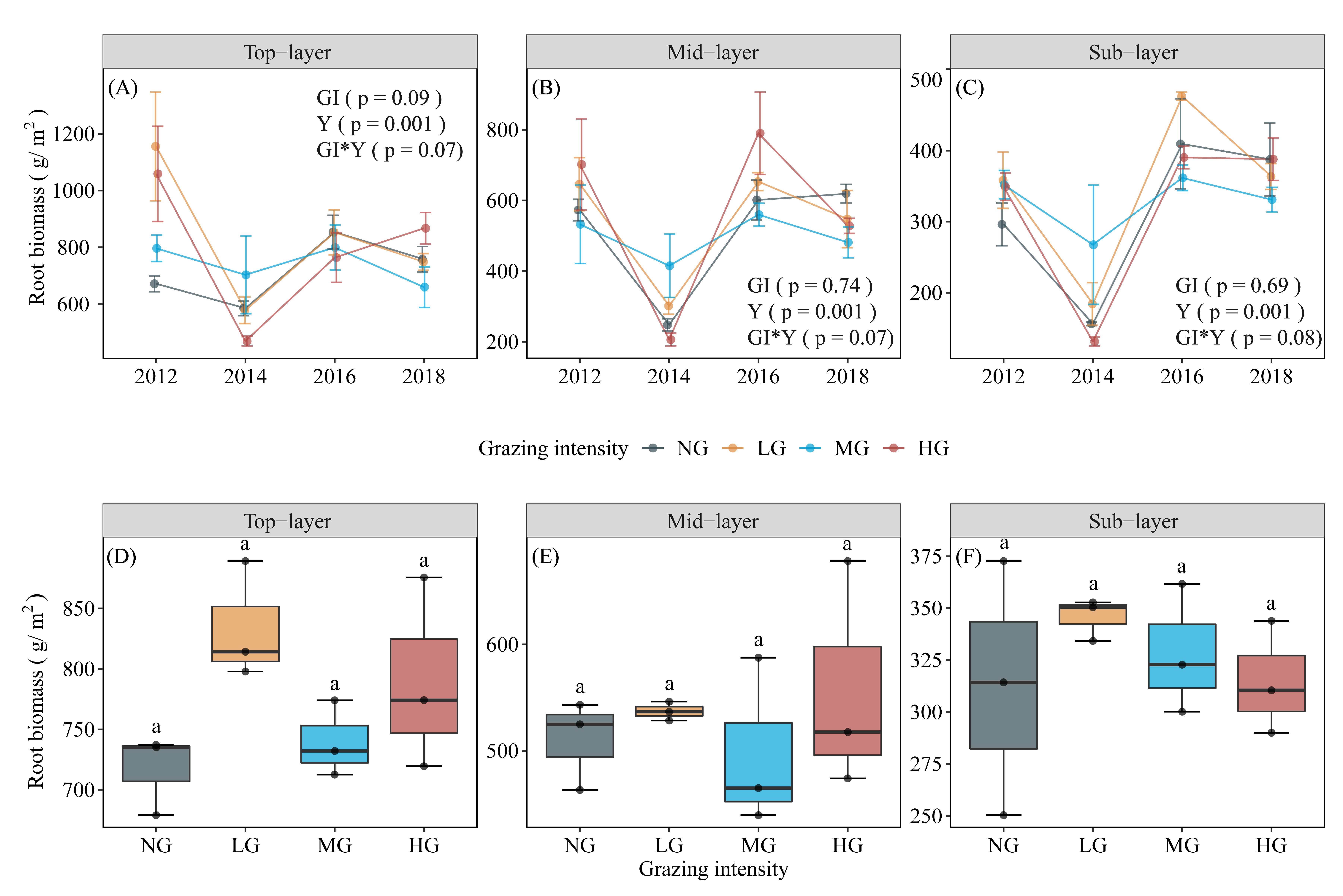
**

**Figure S5.** Root biomass dynamics under different grazing intensities (Mean ± SE, n = 3). Shown are the results of (top-panel) the root biomass dynamics of the (A) top layer, (B) mid-layer and (C) sublayer, with statistics indicating the results from the repeated-measures ANOVA models of grazing intensity, year and their interactions. Tukey’s-range test (bottom-panel) were used to examine differences among different grazing intensities (D: top layer; E: mid-layer and F: sublayer), with lowercase letters represent significant differences among grazing treatments (p < 0.05). Key: NG = no grazing, LG = light grazing intensity, MG = medium grazing intensity, HG = heavy grazing intensity.


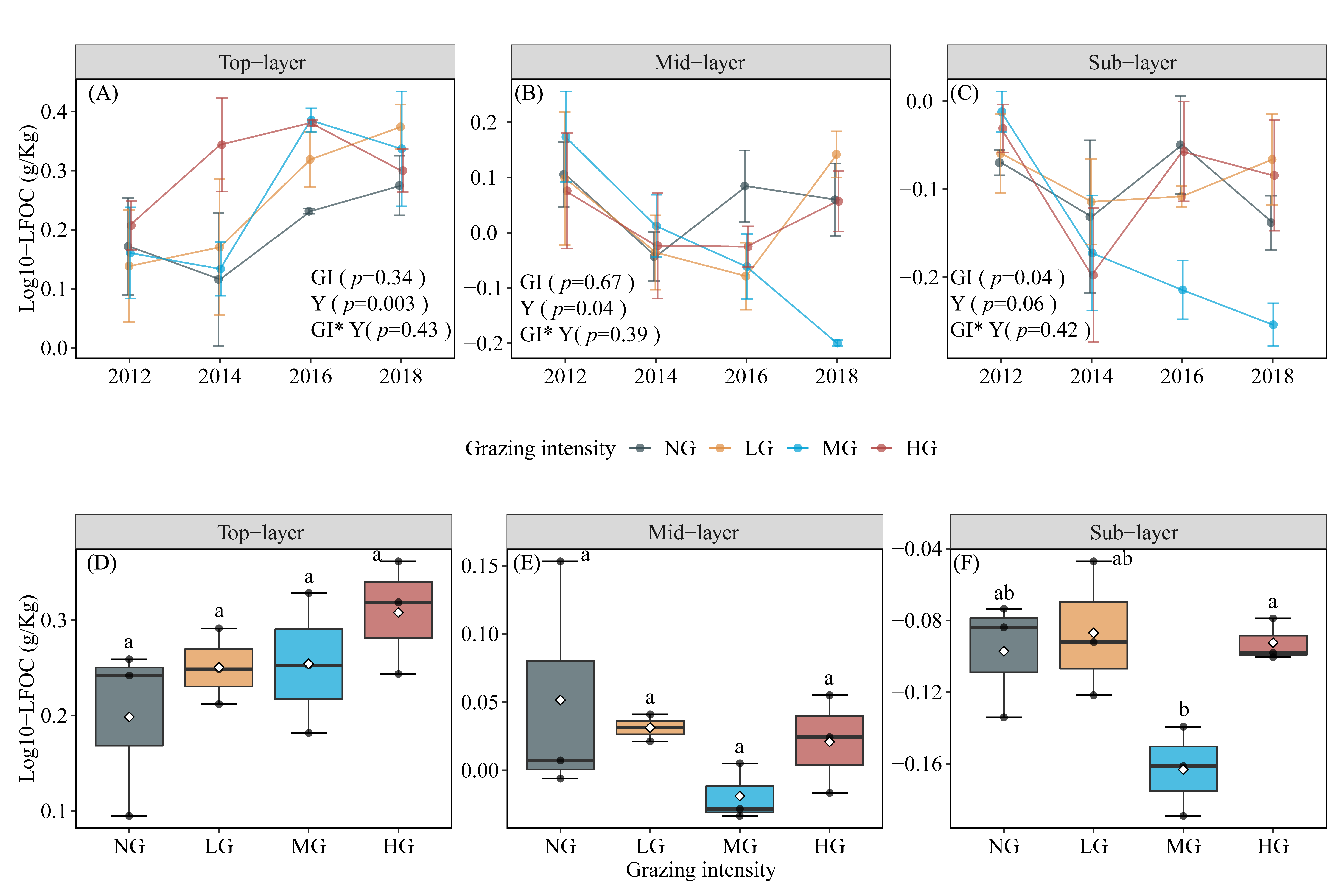


**Figure S6.** Light fraction organic carbon (LFOC) dynamics under different grazing intensities (Mean ± SE, n = 3). Shown are the results of (top-panel) the LFOC dynamics of the (A) top layer, (B) mid-layer and (C) sublayer, with statistics indicating the results from the repeated-measures ANOVA models of grazing intensity, year and their interactions. Tukey’s-range test (bottom-panel) were used to examine differences among different grazing intensities (D: top layer; E: mid-layer and F: sublayer), with lowercase letters represent significant differences among grazing treatments (p < 0.05). Key: NG = no grazing, LG = light grazing intensity, MG = medium grazing intensity, HG = heavy grazing intensity.


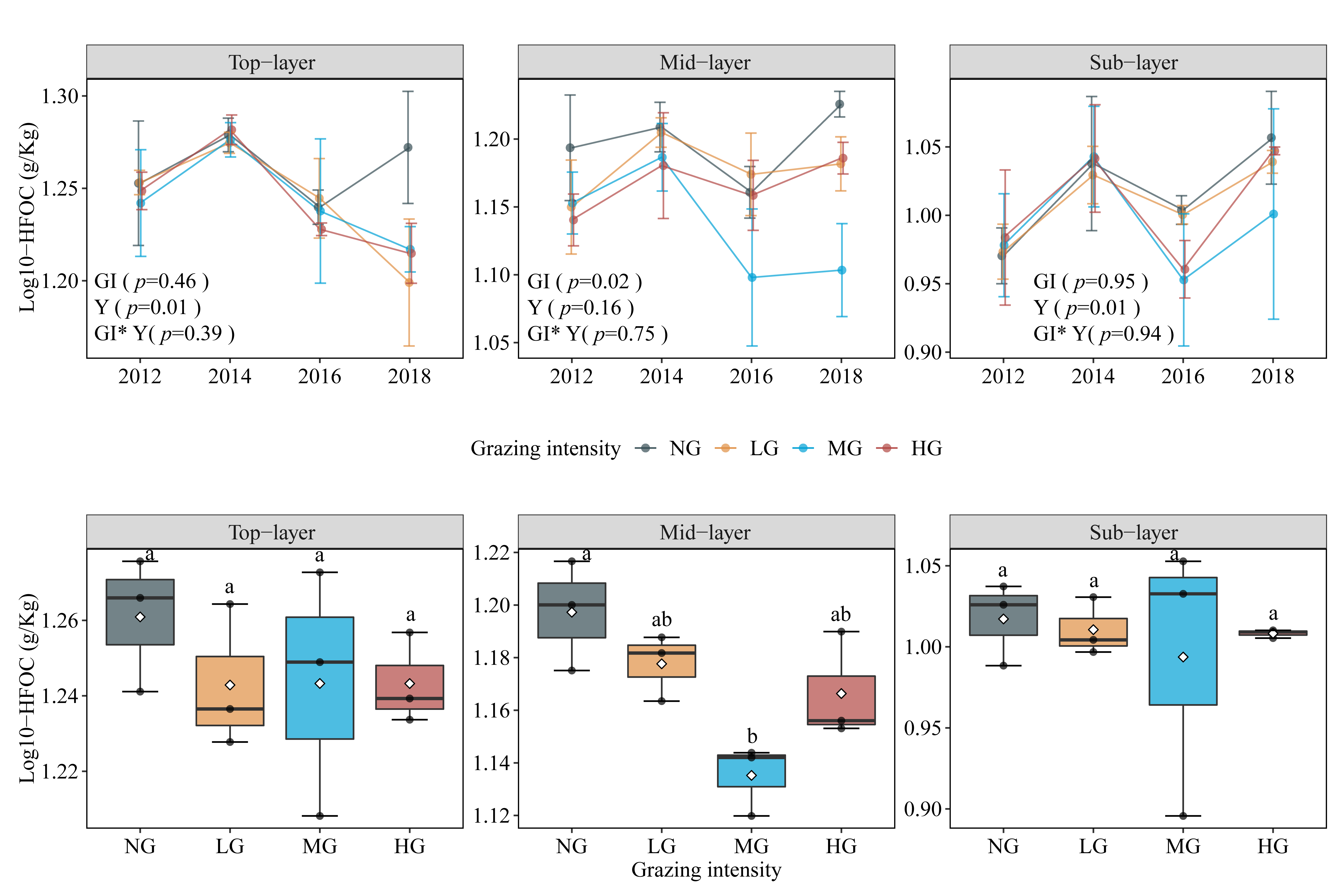


**Figure S7.** Heavy fraction organic carbon (HFOC) dynamics under different grazing intensities (Mean ± SE, n = 3). Shown are the results of (top-panel) the HFOC dynamics of the (A) top layer, (B) mid-layer and (C) sublayer, with statistics indicating the results from the repeated-measures ANOVA models of grazing intensity, year and their interactions. Tukey’s-range test (bottom-panel) were used to examine differences among different grazing intensities (D: top layer; E: mid-layer and F: sublayer), with lowercase letters represent significant differences among grazing treatments (p < 0.05). Key: NG = no grazing, LG = light grazing intensity, MG = medium grazing intensity, HG = heavy grazing intensity.**
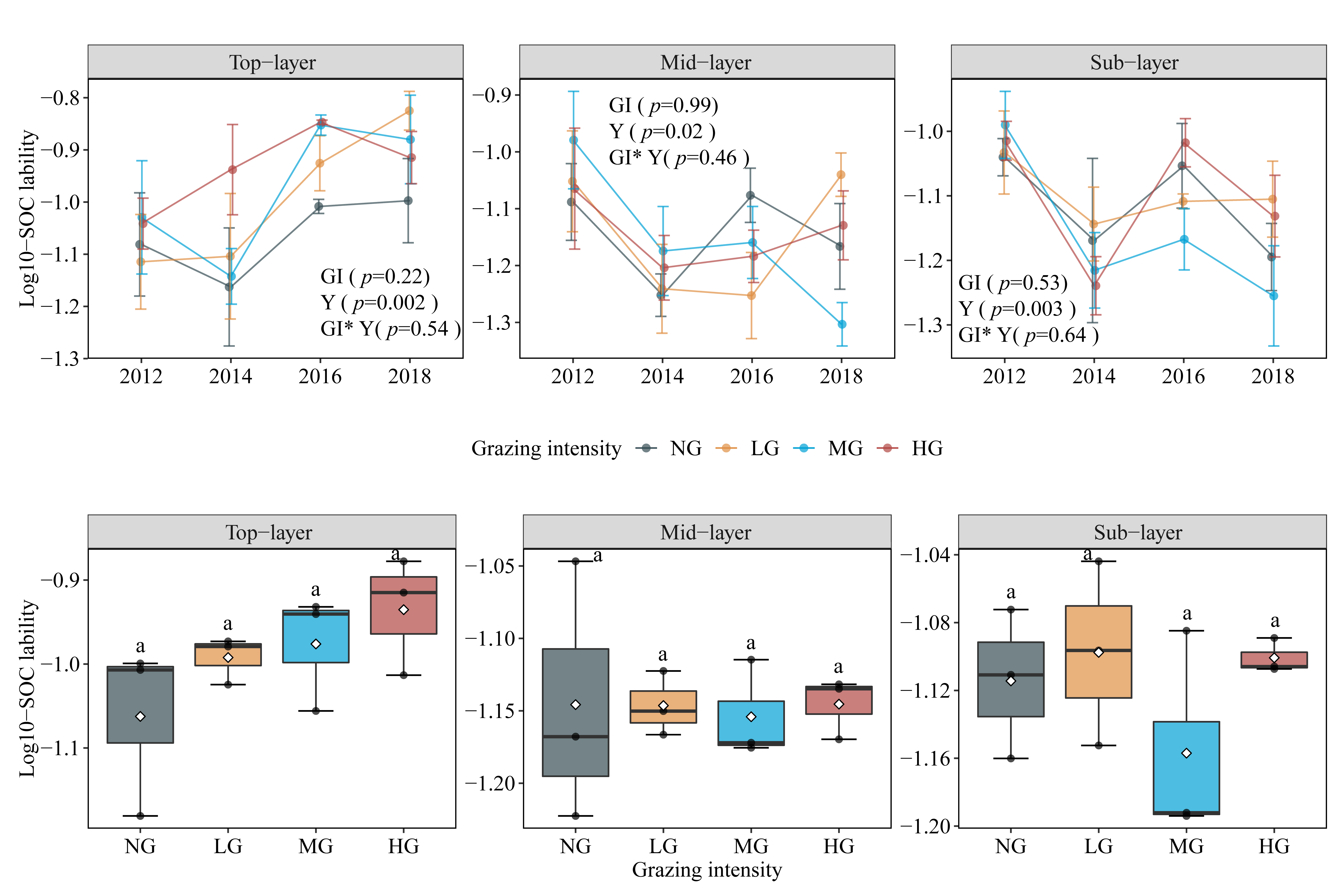
**

**Figure S8.** Soil organic carbon lability (SOC lability) dynamics under different grazing intensities (Mean ± SE, n = 3). Shown are the results of (top-panel) the SOC lability dynamics of the (A) top layer, (B) mid-layer and (C) sublayer, with statistics indicating the results from the repeated-measures ANOVA models of grazing intensity, year and their interactions. Tukey’s-range test (bottom-panel) were used to examine differences among different grazing intensities (D: top layer; E: mid-layer and F: sublayer), with lowercase letters represent significant differences among grazing treatments (p < 0.05). Key: NG = no grazing, LG = light grazing intensity, MG = medium grazing intensity, HG = heavy grazing intensity.


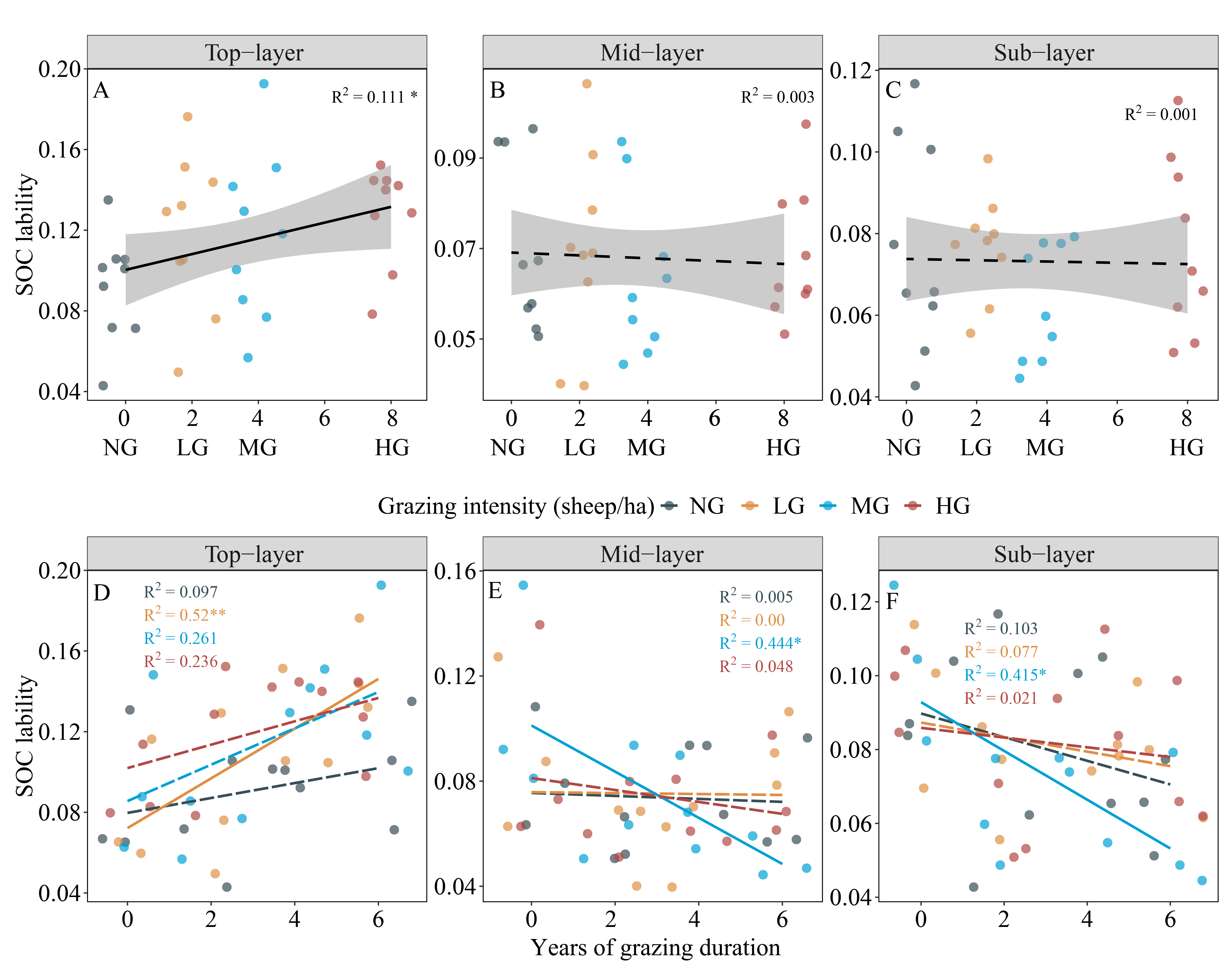


**Figure S9.** Soil organic carbon lability (SOC lability) response to grazing intensity (A-C) and grazing duration (D-F) in different soil layers (A, D: top layer; B, E: mid-layer and C, F: sublayer). The solid and dashed lines indicate significance and non-significance, respectively. Significant levels are: *: *p* < 0.05; ** *p* < 0.01. NG = no grazing, LG = light grazing intensity, MG = medium grazing intensity, HG = heavy grazing intensity.


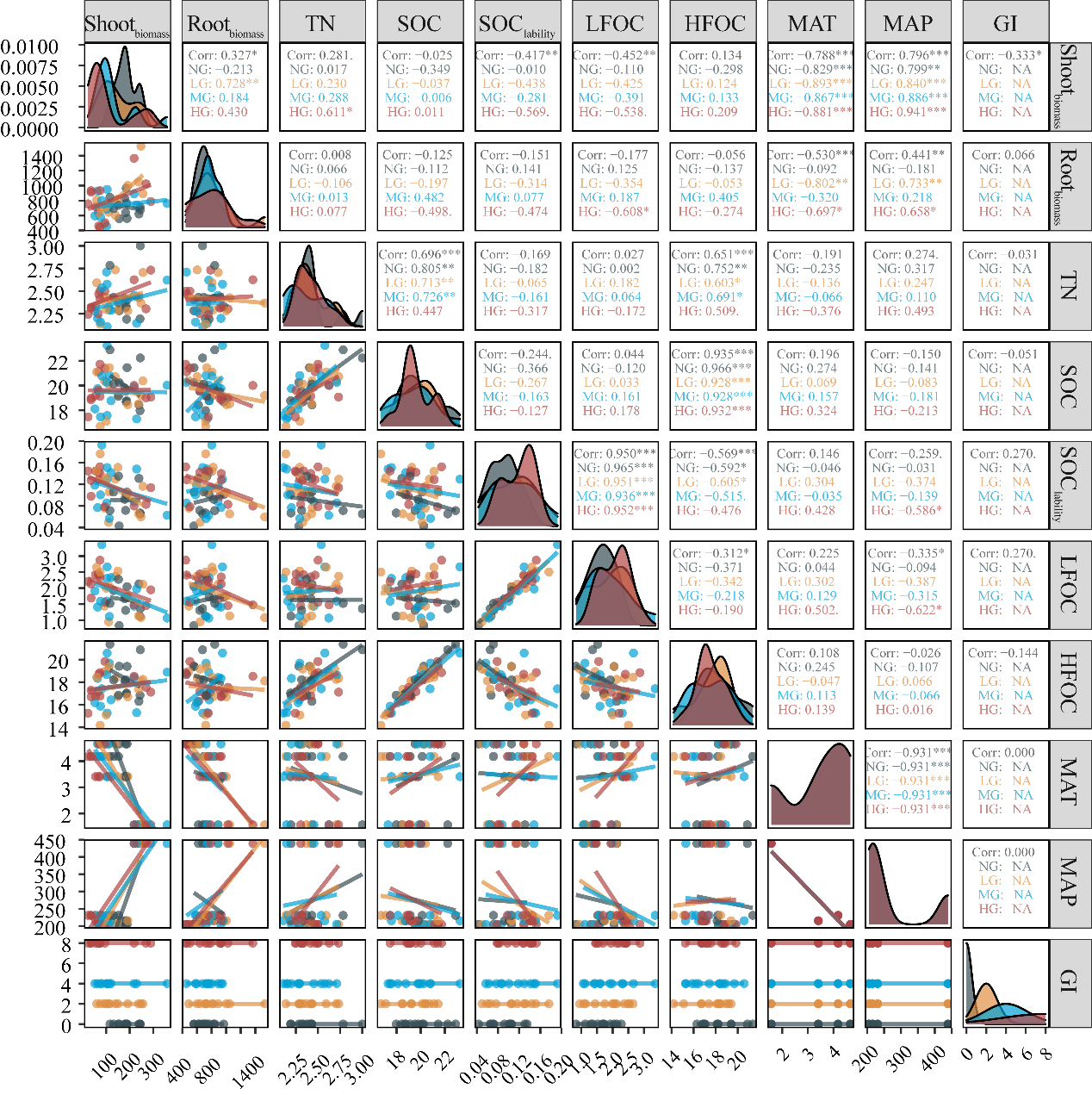


**Figure S10.** Pearson’s correlation coefficients between shoot biomass, root biomass, TN, SOC, SOC lability, LFOC, HFOC, MAT, MAP and GI in the top layer (0 – 10 cm) under different grazing intensities. Significance levels are.: *p* < 0.10; *: *p* < 0.05; **: *p* < 0.01; ***: *p* < 0.001. SOC = soil organic carbon; TN = total nitrogen; NG = no grazing; LG = light grazing intensity; MG = medium grazing intensity; HG = heavy grazing intensity.


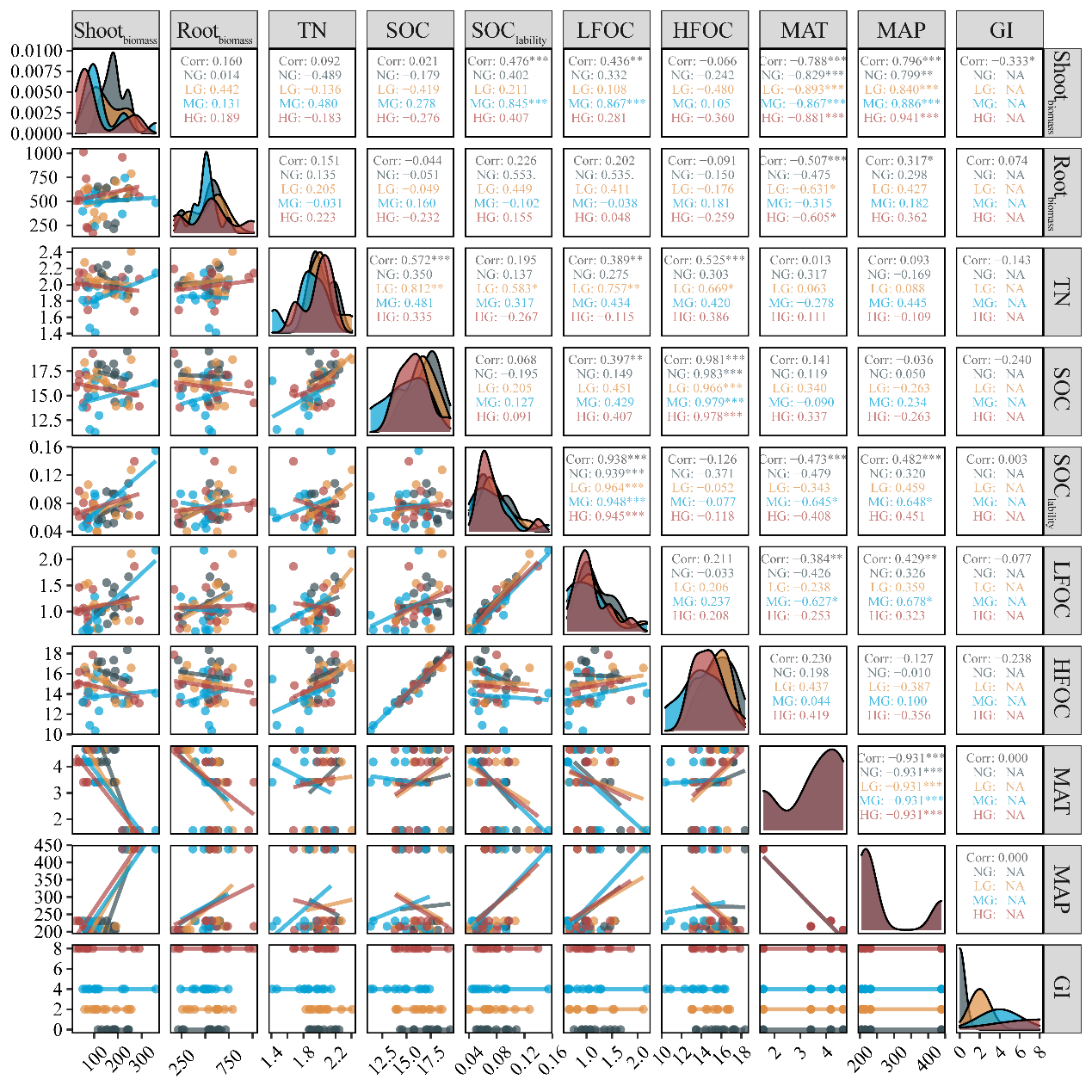


**Figure S11.** Pearson’s correlation coefficients between shoot biomass, root biomass, TN, SOC and SOC lability in the mid-layer (10 – 30 cm) under different grazing intensities. Significance levels are.: *p* < 0.10; *: *p* < 0.05; **: *p* < 0.01; ***: *p* < 0.001. SOC = soil organic carbon; TN = total nitrogen; NG = no grazing; LG = light grazing intensity; MG = medium grazing intensity; HG = heavy grazing intensity.

**
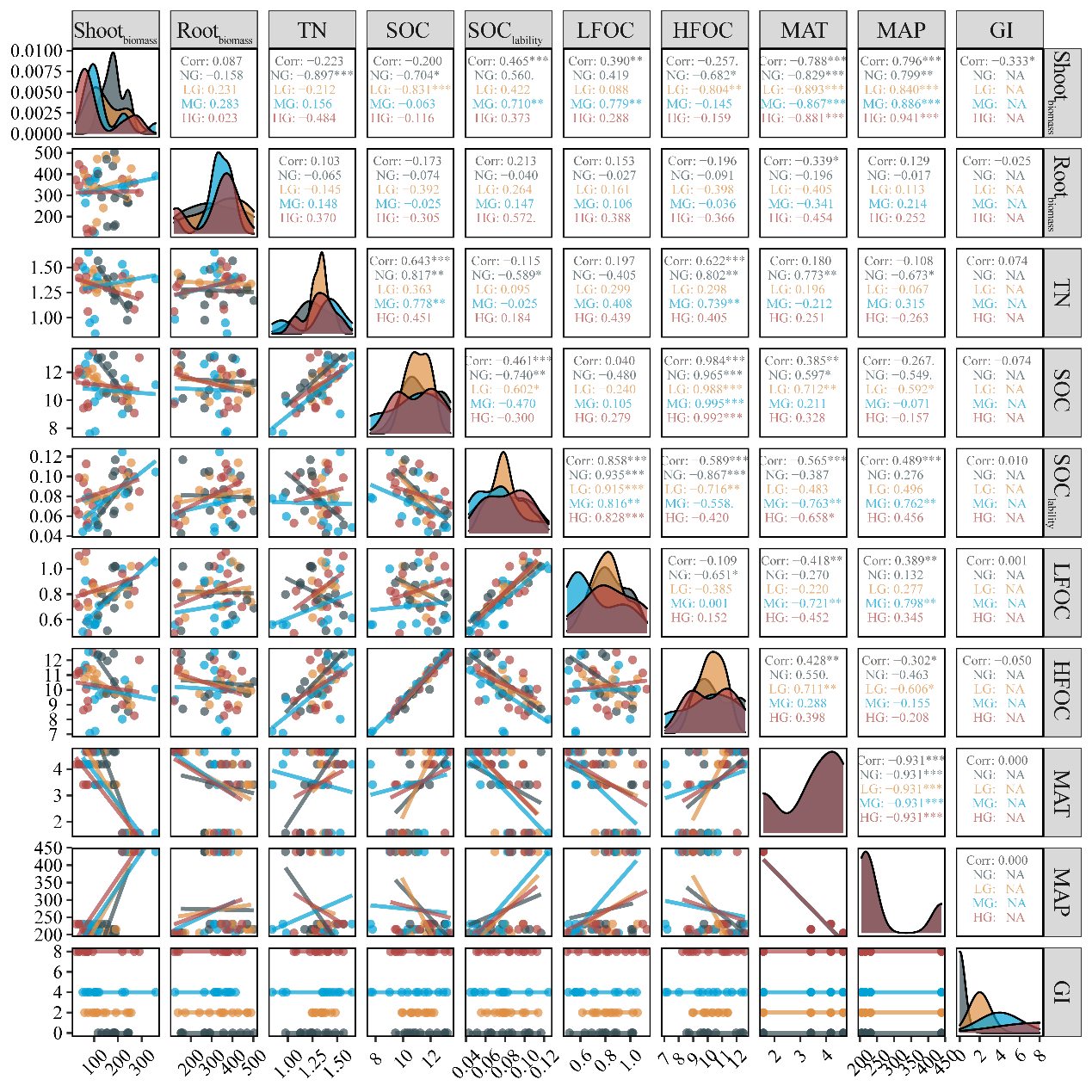
**

**Figure S12.** Pearson’s correlation coefficients between shoot biomass, root biomass, TN, SOC and SOC lability in the sublayer (30 – 50 cm) under different grazing intensities. Significance levels are.: *p* < 0.10; *: *p* < 0.05; **: *p* < 0.01; ***: *p* < 0.001. SOC = soil organic carbon; TN = total nitrogen; NG = no grazing; LG = light grazing intensity; MG = medium grazing intensity; HG = heavy grazing intensity.

**
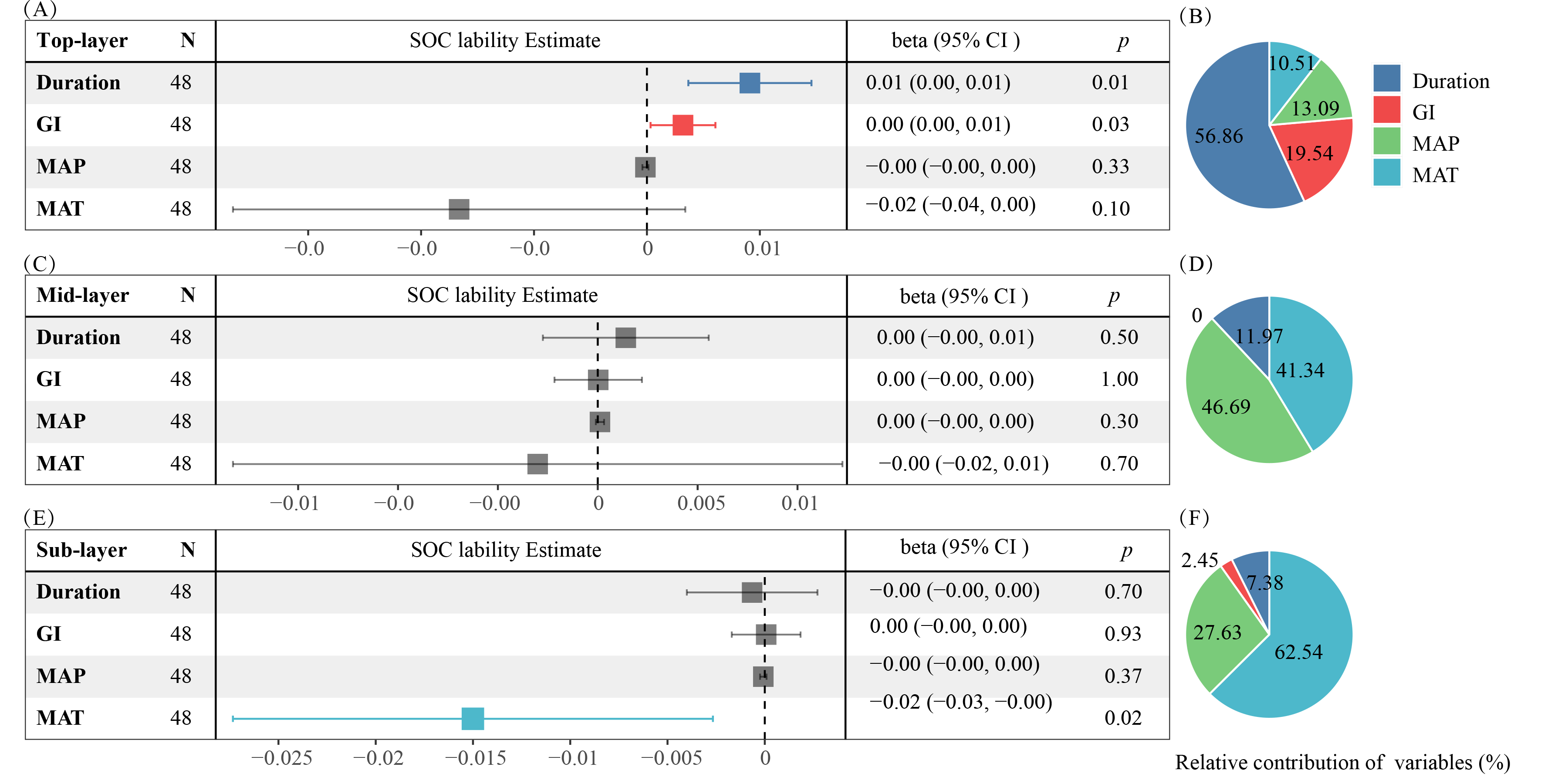
**

**Figure S13.** Standardized SOC lability estimates (A, C and E) and relative contributions (B, D and F) of multiple predictors of the ordinary least squares model for organic carbon in different soil layers (A-B: top layer; C-D: mid-layer and E-F: sublayer) of a typical semiarid grassland in Inner Mongolia. The standardized effect sizes are shown with their 95% confidence intervals, and relative contributions (variance explained by each predictor variable) are assessed using the mean ranking method (lmg indicator in the *relaimpo* R package). Significant differences are indicated by coloured square dots (*p* < 0.05). Duration = years of grazing duration; GI = grazing intensity; MAP = mean annual precipitation; MAT = Mean annual temperature; SOC = soil organic carbon.

**
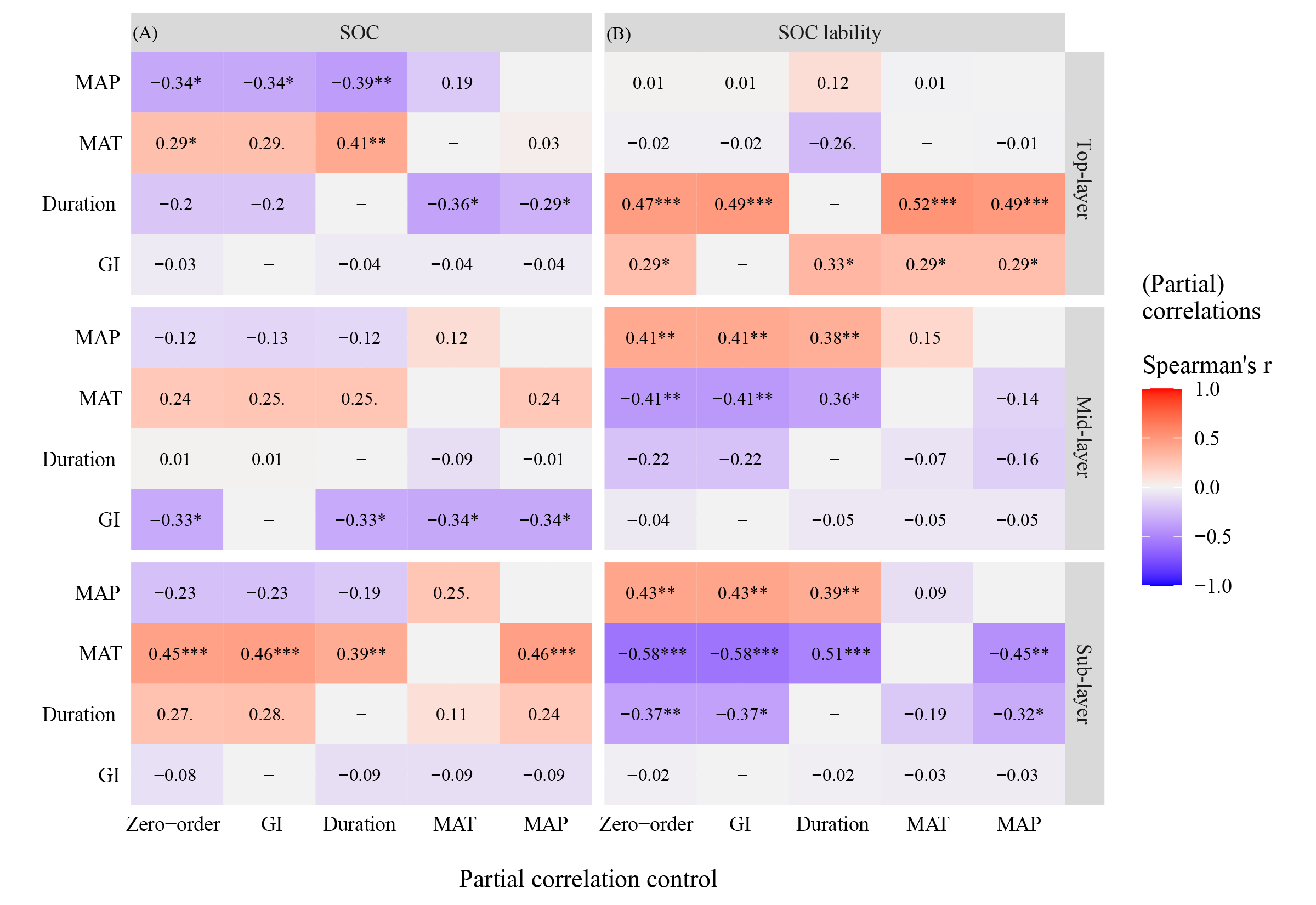
**

**Figure S14.** Partial correlation between SOC (A) and SOC lability (B) with years and grazing effects in different soil layers (Spearman's r). The intensity of colours and numbers indicate the strength of the correlation. Significance levels are marked as follows.: *p* < 0.10; *: *p* < 0.05; ** *p* < 0.01 and ***: *p* < 0.001. Duration = years of grazing duration; GI = grazing intensity; MAP = mean annual precipitation; MAT = mean annual temperature; SOC = soil organic carbon.


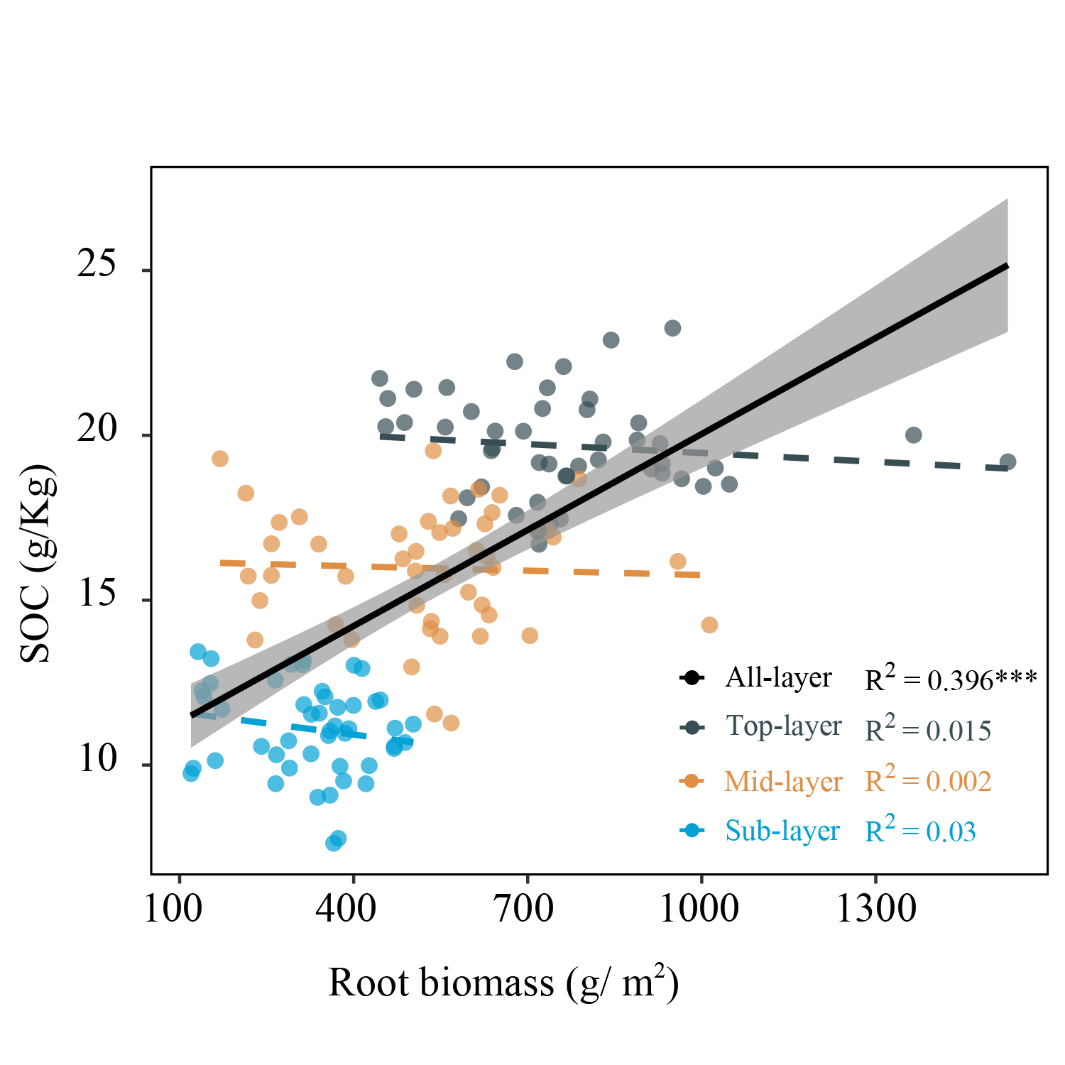


**Figure S15.** Response of soil organic carbon to root biomass in different soil layers. The solid and dashed lines indicate significance and non-significance, respectively. Significant levels are: *: *p* < 0.05; ** *p* < 0.01, *** *p* < 0.001. All-layer: 0-50 cm, top layer: 0-10 cm, mid-layer: 10-30 cm, sublayer: 30-50 cm.


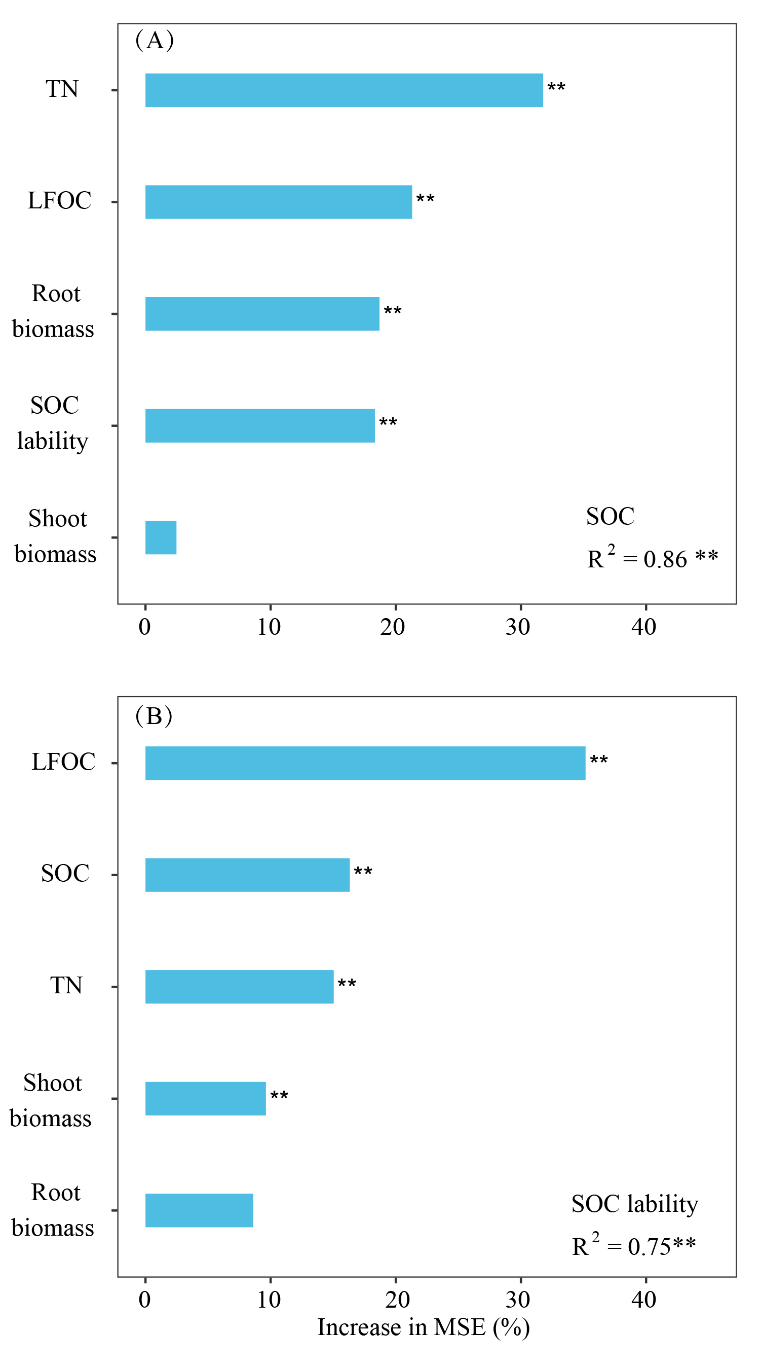


**Figure S16.** The major drivers of SOC (A) and SOC lability (B). The figure illustrates the importance of the five significant factors (analysed by the random forest model) for soil organic carbon and organic carbon stability. R^2^ is the percentage of variance explained by the five variables for SOC and SOC lability. MSE is the mean square error. Significance levels are: *: *p* < 0.05; **: *p* < 0.01. SOC = soil organic carbon; LFOC = light fraction organic carbon; TN = total nitrogen.

**Table S1.** Standardized SOC and SOC lability estimates of multiple predictors of the linear ridge regression model in different soil layers of a typical semiarid grassland in Inner Mongolia. Significant differences are indicated by bold font (*p* < 0.05). Duration = years of grazing duration; GI = grazing intensity; MAP = mean annual precipitation; MAT = mean annual temperature; SOC = soil organic carbon.

| Soil layer | Variable | SOC | | | SOC lability | | |
| --- | --- | --- | --- | --- | --- | --- | --- |
|  |  | Estimate | *T* value | *p* | Estimate | *T* value | *p* |
| Top-soil | Duration | **-3.67** | **2.526** | **0.01** | **0.10** | **4.03** | **< 0.001** |
|  | GI | -0.40 | 0.32 | 0.75 | **0.05** | **2.20** | **0.01** |
|  | MAP | -1.37 | 0.99 | 0.32 | 0.04 | -0.02 | 0.34 |
|  | MAT | 2.18 | 1.56 | 0.12 | -0.03 | -0.03 | 0.12 |
| Mid-soil | Duration | -0.80 | 0.79 | 0.43 | -0.001 | 0.02 | 0.99 |
|  | GI | -1.80 | 1.68 | 0.09 | 0.001 | 0.02 | 0.98 |
|  | MAP | 0.20 | 0.27 | 0.78 | **0.04** | **2.98** | **0.003** |
|  | MAT | 1.47 | 1.78 | 0.07 | **-0.03** | **2.53** | **0.01** |
| Sub-soil | Duration | 0.30 | 0.34 | 0.74 | -0.01 | 0.49 | 0.62 |
|  | GI | -0.47 | 0.53 | 0.59 | 0.001 | 0.08 | 0.93 |
|  | MAP | -0.18 | 0.26 | 0.79 | 0.02 | 1.37 | 0.17 |
|  | MAT | **2.24** | **3.10** | **0.002** | **-0.05** | **3.77** | **< 0.001** |
